# Regulation of miR163 and its targets in defense against *Pseudomonas syringae* in *Arabidopsis thaliana*

**DOI:** 10.1038/srep46433

**Published:** 2017-04-12

**Authors:** Hiu Tung Chow, Danny W-K. Ng

**Affiliations:** 1 Department of Biology, Hong Kong Baptist University, Kowloon Tong, Hong Kong, China; 2 The Partner State Key Laboratory of Agrobiotechnology, The Chinese University of Hong Kong, Shatin, Hong Kong, China

## Abstract

Small RNAs are important regulators for a variety of biological processes, including leaf development, flowering-time, embryogenesis and defense responses. miR163 is a non-conserved miRNA and its locus has evolved recently through inverted duplication of its target genes to which they belong to the SABATH family of related small-molecule methyltransferases (MTs). In *Arabidopsis thaliana*, previous study demonstrated that miR163 accumulation was induced by alamethicin treatment, suggesting its roles in defense response pathways. Enhanced resistance against *Pseudomonas syringae* pv. *tomato (Pst*) was observed in the *mir163* mutant, whereas transgenic lines overexpressing miR163 showed increase sensitivity to *Pst*, suggesting that miR163 is a negative regulator of defense response. Elevated level of miR163 and its targets in *A. thaliana* were observed upon *Pst* treatment, suggesting a modulating relationship between miR163 and its targets. In addition, miR163 and histone deacetylase were found to act cooperatively in mediating defense against *Pst.* Transgenic plants overexpressing miR163-resistant targets suggested their different contributions in defense. Results from this study revealed that the stress-inducible miR163 and its targets act in concert to modulate defense responses against bacterial pathogen in *A. thaliana*.

Small RNAs, including microRNAs (miRNAs), small interfering RNAs (siRNAs) and trans-acting siRNAs (tasiRNAs) are non-coding RNA molecules that mediate gene silencing through guided DNA methylation, transcript cleavage or translation repression^1^. It is becoming evident that small RNAs play important roles in plant-microbe interactions^2^ and accumulation of miRNAs are tightly regulated under stress conditions^3^,^4^,^5^. In *Arabidopsis*, virulent *Pseudomonas syringae* DC3000 infection was found to suppress the expression of miR393 that targets auxin receptors^6^. Enhanced sensitivity to *P. syringae* DC3000 *hrcC*- was observed in small RNA biogenesis mutants, *dcl1* and *hen1*, indicating that RNA silencing are involved in plant resistance to bacteria^7^. Plant *MIRNA* genes undertake relatively frequent birth and death, with only a subset being preserved through integration into existing regulatory networks for plant development^8^. miRNAs can be categorized into ancient miRNAs and young miRNAs based on their levels of conservation and their formation during evolutionary history^9^. While ancestral miRNAs are conserved along plant lineages^10^, young miRNA genes are more specific and some are originated by inverted duplication from its target locus or mutations within inverted repeats^11^,^12^. Recent studies have begun to elucidate the mechanism for *de novo* generation of non-conserved miRNAs that function in various stress responsive pathways. For an example, a novel miRNA (miR5090) has been identified as a recently evolved miRNA through inverse duplication of its target, *ALKENYL HYDROXALKYL PRODUCING2 (AOP2*). miR5090 is upregulated upon nitrogen starvation and its expression is negatively correlated with *AOP2* accumulation^13^.

miR163 is an *Arabidopsis*-specific miRNA that appears relatively recent in evolutionary time^10^. Different from most typical 21-nt plant miRNAs, miR163 is a non-conserved 24-nt miRNA and its locus has evolved recently by inverted duplication of its target gene that belongs to the SABATH family of small molecules methyltransferases and the *MIR163* locus is located close to a cluster of three SABATH genes on chromosome 1^11^,^14^,^15^. *Pri-miR163* is transcribed by RNA polymerase II and the biogenesis of the mature miR163 is DCL1-dependent through a three-step cleavage of its precursor^11^,^16^. Recent reports also revealed that the 3′ intron of miR163 plays a role in enhancing the biogenesis and production of miR163^17^,^18^. Although the biogenesis of miR163 is well characterized, its regulation and its physiological outcomes through regulation of its target genes are still unknown. Previous study showed that miR163 and its targets (*PXMT1* and *FAMT*) are induced by alamethicin (fungal elicitor) and that *mir163* mutant showed altered secondary metabolite profiles^19^. In this study, we further characterized the roles and regulatory relationship between miR163 and two of its targets (*PXMT1* and *FAMT*) under biotic stresses. Results supported that miR163 is a negative regulator of basal defense against bacterial pathogen. Both miR163 and its targets were induced in response to *P. syringae* infection, suggesting miR163 acts to modulate the target genes expression under stress. In addition to small RNA, epigenetic regulation through histone modifications plays role in coordinating dramatic changes of stress-responsive genes expression^20^,^21^. Many studies have shown that histone deacetylases (HDACs) are involved in stress responses^22^. In *Arabidopsis*, histone deacetylase 19 (HDA19) has been found to interact with several ethylene-responsive factors (ERFs) in mediating abiotic responses^23^. In another study, HDA19 was found to interact with the WRKY transcription factors, WRKY38 and WRKY62, acting as a negative regulator for basal defense^24^. In this study, we found that miR163 accumulation was affected in the *hda19* mutant and it is likely that miR163 and HDA19 act cooperatively in maintaining the basal defense. Direct overexpression of miR163 targets in *A. thaliana* revealed that PXMT1 plays a minor role in defense while FAMT plays a more direct role in defense against the bacterial pathogen. Our findings provide additional insights to the emergence and co-evolution of miRNA and its targets in modulating defense against bacterial pathogens.

## Results and Discussion

### The *mir163* mutant showed enhanced resistance against *Pseudomonas syringae*

**Figure 1:**
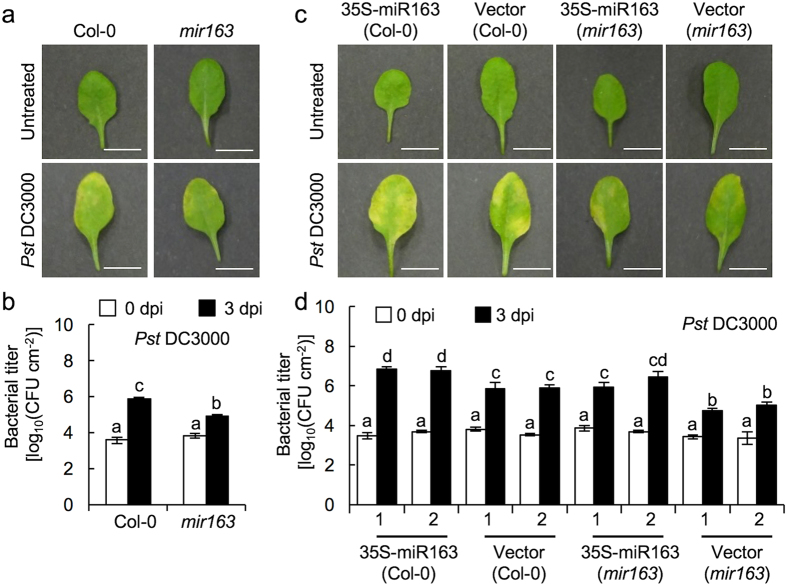
miR163 is involved in defense against *Pseudomonas syringae (Pst*). **(a)** Disease symptoms in mature leaves from 3–4 weeks old *Arabidopsis thaliana* (Col-0 and the *mir163* mutant; CS879797) before inoculation (untreated) and at 3 days post-inoculation (3 dpi) with virulent *Pseudomonas syringae* pv. *tomato* DC3000 (*Pst* DC3000). Inoculation was performed using 5 × 10^5^ cfu/mL (OD_600_ = 0.001) of bacteria through syringe infiltration. Scale bar = 1 cm. **(b)** Bacterial growth in leaves were determined at 0 and 3 dpi. Error bars indicate the standard deviation from 3 replicates. Same letters denote no statistical differences among means from the replicates as calculated by ANOVA with Tukey-Kramer *post hoc* test (α = 0.05). **(c)** Disease symptoms of transgenic lines overexpressing the *pri-miR163* in Col-0 and the *mir163* mutant before inoculation (untreated) and at 3 dpi with *Pst*, respectively. Disease symptoms of the corresponding empty vector control line were showed. Scale bar = 1 cm. **(d)** Bacterial growth analysis in transgenic lines inoculated with *Pst* at 0 and 3 dpi. Bacterial treatment was performed as in **(b)**. Same letters denote no statistical differences among means from three biological replicates as calculated by ANOVA with Tukey-Kramer *post hoc* test (α = 0.05).

It has been showed that miR163 and its targets are potentially involved in plant defense^19^. To further characterize this possible role of miR163, we tested if miR163 and its targets are responsive to the bacterial pathogen, *Pseudomonas syringae* pv. *tomato* DC3000 (*Pst* DC3000) (Fig. 1). Upon *Pst* DC3000 infection, chlorotic symptoms were visible at 3 dpi in the wild type (Col-0) and the *mir163* mutant (Fig. 1a). However, increased resistance to *Pst* DC3000 infection was detected in the *mir163* mutant when compared to Col-0 (Fig. 1b). In a reciprocal experiment, transgenic lines overexpressing the *pri-miR163* under the control of the *CaMV 35S* promoter were assayed for their resistance against *Pst* infection (Fig. 1c to d). Although increased sensitivities toward the bacterial pathogens were observed in transgenic lines overexpressing *pri-miR163* in the *mir163* mutant, the appearance of chlorotic symptoms was not as obvious when compared to that in Col-0 (Fig. 1c). Overall, the *pri-miR163* overexpressors showed 8.18- to 9.23-fold increase in the intercellular bacterial titer within the inoculated leaf tissues when compared to that in the transgenic lines containing the empty vector in Col-0 background. In the *mir163* mutant, overexpression of *pri-miR163* attenuated its resistance against *Pst* DC3000 when compared to the corresponding transgenic lines containing the empty vector. Therefore, these data suggested a negative-regulatory role of miR163 in mediating defense against the bacterial pathogen.

### *P. syringae* induced miR163 expression and accumulation

**Figure 2:**
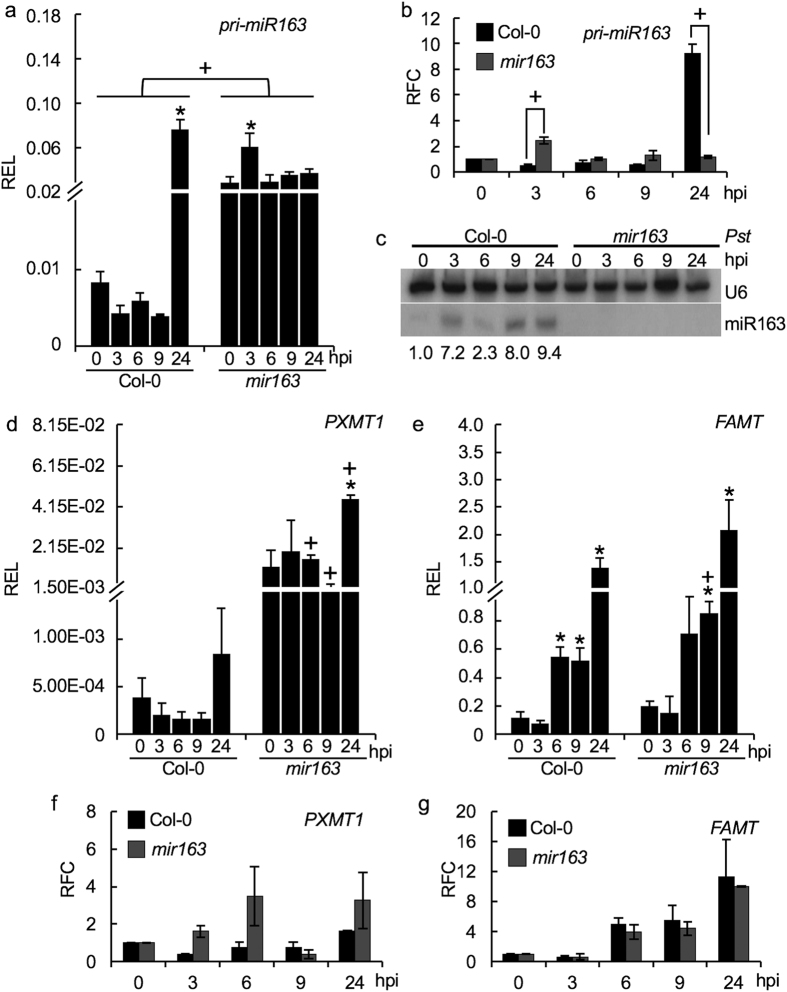
*Pst* induced expression of miR163 and its targets. **(a,b)** Time course expression of *pri-miR163* in Col-0 and the *mir163* mutant after dipping treatment with 2 × 10^8^ cfu/mL (OD_600_ = 0.4) of *Pst* DC3000. Infected leaves from 4 plants were collected at the indicated hours post-inoculation (hpi) for RNA extraction and gene expression analyses. (**a**) The relative expression level (REL) of *pri-miR163* was calculated using *EF1α* as a control. (**b**) Upon induction, the relative fold change (RFC) of *pri-miR163* at different time point was compared to that at 0 hpi. Values are mean ± standard error (n = 3). **(c)** Temporal miR163 accumulation at 0, 3, 6, 9 and 24 hpi in Col-0 and the *mir163* mutant upon infection with *Pst* DC3000 were detected using small RNA gel blot analyses. The corresponding U6 signals (endogenous controls) were detected in the same blot. Densitometric analysis was performed using ImageJ software and the miR163 signals were normalized against U6. The relative fold change of miR163 was showed at the bottom. Experiments were performed twice with similar results. **(d–g)** Expression of two miR163 targets, *PXMT1* (**d**) and *FAMT* **(e)**, in mature leaves of Col-0 and the *mir163* mutant before (0) and at 3, 6, 9, 24 hpi with *Pst* DC3000. The relative expression level (REL) of *PXMT1* and *FAMT* was calculated using *EF1α* as a control. **(f,g)** Relative fold change (RFC) of the targets at various time points upon *Pst* treatment was compared against 0 hpi. Values are mean ± standard error (n = 3). Asterisks indicate significant differences between 0 hpi and the indicated time using Student’s *t*-test (P < 0.05). “+” signs indicate significant differences (Student’s *t-*test; P < 0.05) between Col-0 and the *mir163* at the indicated time point.

Having established that miR163 plays a role in plant defense, we further analyzed *MIR163* expression upon inoculation with *Pst* DC3000 in *A. thaliana*. The level of *pri-miR163* was quantified at different time intervals up to 24 hours post-inoculation (hpi) with the pathogen (Fig. 2). In the wild type *A. thaliana*, the steady-level of *pri-miR163* was similar and remained low at 3, 6 and 9 hpi when compared to 0 dpi, but showed a significant increase at 24 hpi upon the bacterial challenged (Fig. 2a). It has been showed that expression of dicer-like 1 (DCL1), a ribonuclease III enzyme involved in miRNA processing, is induced upon viral infection^25^. It is possible that the general low level of *pri-miR163* detected at 3, 6, and 9 hpi is due to an increase in processing of the *pri-miR163* during the early stage of infection. However, noted that such decreases are insignificant when compared to 0 hpi and could simply attribute to a normal variation of expression. In the *mir163* mutant, the T-DNA is inserted at the mature miR163 region, hence blocking the processing of *pri-miR163* by DCL1^16^. Although an elevated level of *pri-miR163* was detected in the *mir163* when compared to Col-0, no further increase in the *pri-miR163* accumulation was detected when challenged by *Pst* except at 3 hpi (Fig. 2a). Comparing Col-0 and the *mir163*, the relative fold change of *pri-miR163* accumulation was higher in the *mir163* at 3 hpi, suggesting an impeded processing of the induced *pri-miR163* (Fig. 2b). Using small RNA blot, a rapid induction of miR163 accumulation in Col-0 was detected at 3 hpi upon the *Pst* treatment (Fig. 2c). The increase in miR163 accumulation appeared to be limited at 6 hpi but showed an increase again at 9 hpi and 24 hpi after the *Pst* treatment. Moreover, while a 9-fold increase in the *pri-miR163* accumulation was detected in Col-0 at 24 hpi, there was only a small fold change (~1.1) increase in the mature miR163 accumulation between 9 and 24 hpi. Therefore, these differential expression and accumulation of *pri-miR163* and miR163 upon *Pst* infection have provided evidence that the expression and biogenesis of miR163 are affected by biotrophic pathogens^17^,^18^. In contrast, miR163 accumulation remained low under the control (MgCl_2_) treatment (see Supplementary Fig. S1). As expected, impeded biogenesis of miR163 due to T-DNA insertion in the *mir163* mutant has abolished the accumulation of mature miR163 in the mutant.

### Dynamic regulation of miR163 and its targets in response to pathogen attacks

In *Arabidopsis*, the *MIR163* locus evolved through duplication from its target gene locus and two miR163 targets, *PXMT1* and *FAMT* have been identified^11^,^19^,^26^. Therefore, we analyzed the expression dynamics of its targets in a time course experiment. Using qRT-PCR, the overall transcripts accumulation upon *Pst* treatment was compared between Col-0 and the *mir163* mutant. This will enable us to test if the altered expressions of these targets are associated with the observed *Pst* resistance phenotype in the *mir163* when compared to the Col-0. In addition, the expression fold changes of the targets between the lines were compared to determine the regulatory role of miR163 on the targets. Under normal growth condition, *PXMT1* expression is repressed in Col-0 and accumulated at a higher level in the *mir163* mutant (Fig. 2d). When challenged by *Pst* DC3000, *PXMT1* expression is induced to a higher level at 24 hpi in the *mir163* mutant but not in the Col-0 (Fig. 2d). Similar to *pri-miR163* accumulation, *PXMT1* showed a lower level but insignificant difference in accumulation at 3, 6 and 9 hpi when compared to that at 0 hpi. Such low level of accumulation could reflect the targeting of *PXMT1* by the increased miR163 accumulation or simply a variation due to its low level of accumulation. While *PXMT1* showed an overall high level of accumulation in the *mir163* mutant, no significant fold change increase/decrease of its accumulation was detected upon *Pst* infection when compared to the Col-0 (Fig. 2f). Therefore, this suggested that like miR163, *Pst* treatment is capable of inducing *PXMT1* expression. However, the post-transcriptional control by miR163 plays a more important role in regulating *PXMT1* transcript accumulation rather than the magnitude of *PXMT1* transcriptional activation under stress. Similar to *PXMT1*, expression of *FAMT* is inducible by *Pst* treatment (Fig. 2e). Although, *FAMT* showed a higher basal expression in Col-0 under normal condition, it was induced in both Col-0 (at 6, 9 and 24 hpi) and the *mir163* mutant (at 9 and 24 hpi) (Fig. 2e). In Col-0, a 5-fold increase of *FAMT* accumulation was detected in Col-0 at 6 hpi. Similar folds of induction of *FAMT* were also observed in the *mir163* mutant (Fig. 2g), suggesting a less direct role of miR163 in mediating *FAMT* transcript accumulation. In plant, a range of differential miRNA targeting preference was observed for mRNAs with differential degree of miRNA-target complementarity^27^. miR396 exhibited a strong efficiency in mediating cleavage of its target when the miRNA-target pair is perfectly matched^28^. Interestingly, miR163 and *PXMT1* showed perfect complementarity except a single nucleotide bulge at position 20 of miR163. However, mismatches at positions 8 and 9 were found at the miR163 target site in *FAMT*^11^,^19^. Therefore, such difference in the level of complementarity could lead to different targeting efficiency by miR163, preventing over activation of the target genes, and contributing to the expression difference between the two targets under biotic stresses.

Among the SABATH family of methyltransferases genes, some are inducible (including both miR163 targets) by various stress treatments such as salicylic acid (SA) treatment and wounding^14^. In the presence of exogenous SA, no change in *pri-miR163* expression was observed in Col-0, while the *pri-miR163* expression levels exhibited ~18-fold induction in the *mir163* mutant (see Supplementary Fig. S2a and b). Small RNA blot showed miR163 was indeed induced in Col-0 from a 2-fold induction at 3 hpt to a 6-fold induction at 24 hpt in the presence of exogenous SA (see Supplementary Fig. S2c). Therefore, these data suggested that miR163 is inducible by SA treatment and an efficient processing and biogenesis of miR163 was evident in Col-0 upon the induction (see Supplementary Fig. S2c). In the presence of the SA-induced miR163, both *PXMT1* and *FAMT* are expected to be down-regulated. However, expression of *PXMT1* and *FAMT* are significantly up-regulated in the presence of exogenous SA (see Supplementary Fig. S3), suggesting SA-dependent induction of the two targets and such induction overcame the negative regulation by the induced miR163. Consistent with this finding, *PXMT1* and *FAMT* accumulated at a higher level in the *mir163* mutant when compared to the wild type in the presence of SA.

The inverted duplication of miR163 targets resulted in the capture of partial founder gene promoter at the *MIR163* locus^29^. Conserved short sequence at the *MIR163* promoter and its target promoter thus could share similar *cis-*element motifs in controlling their temporal expression in response to environmental stresses. *In silico* analyses of the upstream sequences of the miR163 and the two targets revealed the presence of a variety of putative elicitor and phytohormones responsive elements (see Supplementary Table S1 and Supplementary Methods). In addition, several stress-responsive elements (GT1-binding site, MYB-binding site and W-box) were found to share between these promoters, suggesting their potential role in conferring the stress-responsive expression of miR163 and its targets. To further verify if both *PXMT1* and *FAMT* are inducible by *Pst* DC3000 treatment, the upstream region of *PXMT1* (−1406/ + 68) and *FAMT* (−1124/ + 56) were cloned upstream of a luciferase (LUC) reporter and transformed into *Arabidopsis*, creating ProPXMT1-LUC and ProFAMT-LUC lines for expression analyses. Upon treatment with *Pst* DC3000 at 24 hpi, both reporter lines showed positive LUC expression as revealed by bioluminescence assays (see Supplementary Fig. S4 and Supplementary Methods). Therefore, these data further supported that miR163 and its targets are induced by *Pst*. Future studies using promoter deletion and expression analyses could provide additional insights to their spatial control and regulation under stresses.

### Induction of defense genes expression in the *mir163* mutant

**Figure 3:**
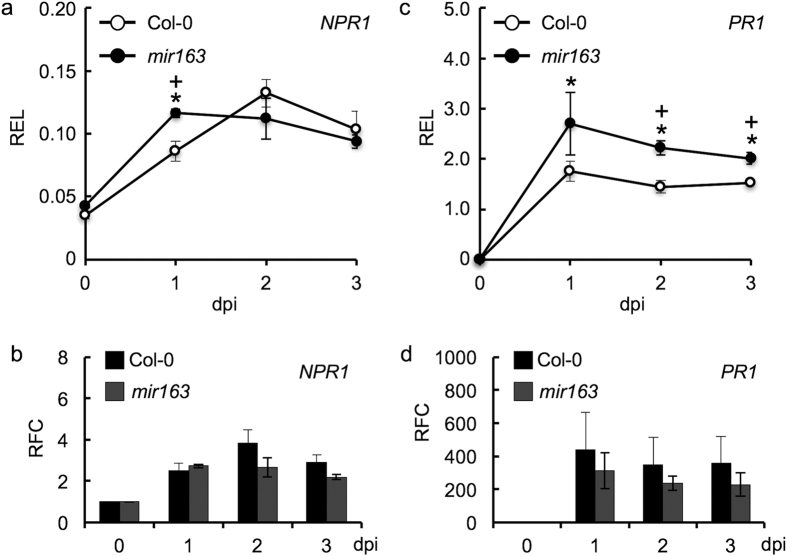
Expression of defense responsive gene in the Col-0 and *mir163* mutant upon *Pst* DC3000 infection. qRT-PCR of *NPR1* **(a,b**) and *PR1* **(c,d)** expression in Col-0 and the *mir163* mutant before (0) and at 1, 2, 3 days post-inoculation (dpi) with the virulent *Pst* DC3000. Inoculation was performed using 5 × 10^5^ cfu/mL (OD_600_ = 0.001) of bacteria through syringe infiltration. The relative expression level (REL) of *NPR1* **(a)** and *PR1* **(c)** was normalized against *EF1α* expression. In addition, the relative fold change (RFC) of *NPR1* **(b)** and *PR1* **(d)** at various time points upon *Pst* treatment was compared against 0 hpi. Values are mean ± standard error (n = 3). Asterisks indicate significant differences between 0 hpi and the indicated time within the genotype using Student’s *t*-test (P < 0.05). “+” signs indicate significant differences (Student’s *t-*test; P < 0.05) between Col-0 and the *mir163* at the indicated time point.

To investigate whether enhanced pathogen resistance in the *mir163* mutant is associated with specific components in the SA-signaling pathway, we examined the expression of two SA-mediated defense markers *NONEXPRESSOR OF PATHOGENESIS-RELATED GENES 1 (NPR1*) and *PATHOGENESIS-RELATED (PR1*) following *Pst* DC3000 inoculation. Upon *Pst* infection, the transcript levels of *NPR1* and *PR1* were elevated in Col-0 and the *mir163* mutant after 1 dpi with 2.2–2.7 folds and 228–313 folds increased in *NPR1* and *PR1* accumulation, respectively (Fig. 3 and see Supplementary Fig. S5). Between Col-0 and the *mir163* mutant, although the transcript level of *NPR1* in the *mir163* mutant was higher at 1 dpi when compared to Col-0 after inoculation with *Pst* DC3000 (Fig. 3a), no difference in *NPR1* expression was observed at 2 and 3 dpi between Col-0 and the *mir163*. In contrast to *NPR1* expression, *PR1* expression level was significantly higher in the *mir163* mutant at 2 and 3 dpi when compared to that in the Col-0 plants (Fig. 3c). Although differential expression of *NPR1* and *PR1* was observed between Col-0 and the *mir163* mutant, the *Pst-*induced expression fold changes of these transcripts are similar between the two genotypes (Fig. 3b and d). Since *mir163* is less susceptible to *Pst* infection when compared to the Col-0, these data suggested that miR163 mutation could enhance R-gene-mediated resistance and components in the SA-signaling pathway indirectly. In addition, expression of the ethylene- and jasmonate-responsive plant defensin, *plant defensin 1.2 (PDF1*.2); and the camalexin biosynthesis gene, *phytoalexin deficient 3 (PAD3*) were analyzed (see Supplementary Fig. S5). Under *Pst* DC3000 treatment, *PAD3* showed similar expression between the Col-0 and the *mir163* mutant (see Supplementary Fig. S5c). In contrast, a lag in the induction of *PDF1.2* was observed in the *mir163* mutant at 6 and 9 hpi (see Supplementary Fig. S5d), suggesting its positive effect on *PDF1.2* expression and its potential involvement in the JA-signaling pathway.

### Cooperate actions of miR163 and histone deacetylase in defense against *Pst* DC3000

**Figure 4:**
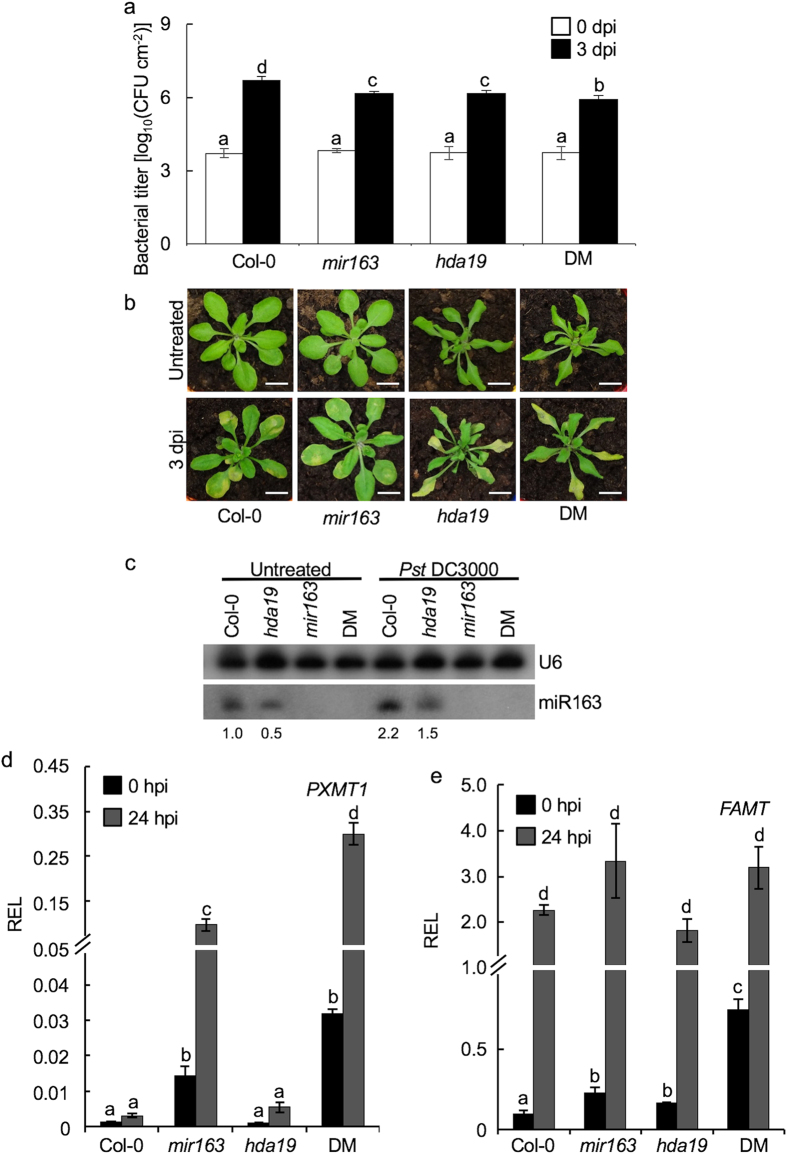
miR163 and *HDAC* act cooperatively in defense against *Pst* DC3000. **(a)** Wild type (Col-0), the *mir163* mutant, the *hda19* mutant (SALK_139445) and homozygous *mir163 hda19* double mutant (DM) were infiltrated with a suspension of *Pst* DC3000 (OD_600_ = 0.001 in 10 mM MgCl_2_; 5 × 10^5^ cfu/mL). Leaves from 4 plants were harvested and bacterial growth in leaves was determined at 0 and 3 days post inoculation (dpi). Error bars indicate the standard deviation from 3 replicates. Same letters denote no statistical differences among means as calculated by ANOVA with Tukey-Kramer *post hoc* test (α = 0.05). Pictures from representative plant were taken at 0 and 3 dpi. Scale bar = 1.5 cm. **(b**) Mature leaves were collected at 0 (untreated) and 24 hours post inoculation (hpi) upon *Pst* DC3000 inoculation for RNA isolation and cDNA synthesis. miR163 accumulation in various plant lines were detected using small RNA gel blot analysis. The corresponding U6 signals were detected in the same blot. Densitometric analysis was performed using ImageJ software and the miR163 signal was normalized against U6. The relative fold differences (bottom) of miR163 were compared against Col-0 at untreated condition. **(c,d)** Expression of *PXMT1* **(c)** and *FAMT* **(d)** were detected using qRT-PCR at 0 and 24 hpi. Values are mean ± standard error (n = 3). Difference in expression between lines at 0 and 24 hpi were calculated using Student’s *t*-test (P < 0.05). Same letters denote no statistical differences among means.

It has been shown that HDA19 is involved in modulating plant defense response through interactions with transcription factors or direct association at stress-responsive gene promoters^24^,^30^. To characterize if genetic interaction exists between miR163 and histone deacetylation in defense signaling, we crossed the *mir163* with the *hda19* mutants, and selected a homozygous *mir163 hda19* double mutant (DM) line for subsequent analyses. Like the *mir163* mutant, the *hda19* mutant showed increased resistance against *Pst* DC3000 and lower bacterial titer was detected in the *hda19* mutant at 3 dpi when compared to the Col-0 (Fig. 4a). In fact, increase disease resistance against *Pst* was found to associate with the loss of *HDA19* activity in *Arabidopsis*^30^. In addition, a slight increase in resistance against *Pst* DC3000 was also detected in the DM line when compared to either of the single mutant. Although both the *hda19* and DM lines showed improved resistance against *Pst* as revealed by the bacterial titers (Fig. 4a), similar chlorotic phenotypes were observed in these lines when compared to the Col-0 (Fig. 4b). Such discrepancy in bacterial titers and chlorotic symptoms observed between the wild type and the *hda19* or DM could be resulted from the fact that HDA19 is a general transcriptional repressor^31^, which could potentially affect the chlorotic phenotype development in the lines upon pathogen infection. In the *hda19* mutant, a decreased basal miR163 accumulation and a lower accumulation of miR163 by *Pst* DC3000 were evident when compared to Col-0 (Fig. 4b; see Supplementary Fig. S6), which is consistent with the observed disease resistance in Fig. 4a. In the *mir163* mutant, increased *PXMT1* accumulation was detected and such increase was more evident in the DM upon *Pst* DC3000 treatment when compared to the Col-0 (Fig. 4c). However, no increase in *PXMT1* accumulation was observed in the *hda19* mutant, suggesting *PXMT1* expression is not directly affected by *HDA19* mutation. Alternatively, it is possible that the miR163 in the *hda19* mutant is sufficient for the downregulation of *PXMT1* transcript upon *Pst* infection. Since *PXMT1* expression is enhanced in the DM line when compared to that in the *mir163* mutant, it suggested that a low induction of miR163 is sufficient to suppress *PXMT1* expression in the *hda19* mutant. Unlike *PXMT1*, basal accumulation of *FAMT* increased in the *hda19* mutant and was further enhanced in the DM compared to either of the single mutant (Fig. 4d), suggesting HDA19 act to repress *FAMT* expression. However, similar induction of *FAMT* upon *Pst* DC3000 treatment was observed in the wild-type, single mutants and the DM lines (Fig. 4d). Overall, these data suggested a cooperative interaction between miR163 and histone deacetylase in modulating *FAMT* induction during *Pst* infection and that histone deacetylation contributed as a part of negative regulation of *FAMT* expression.

### Histone modifications change at the *MIR163* and its target loci upon *Pst* challenge

**Figure 5:**
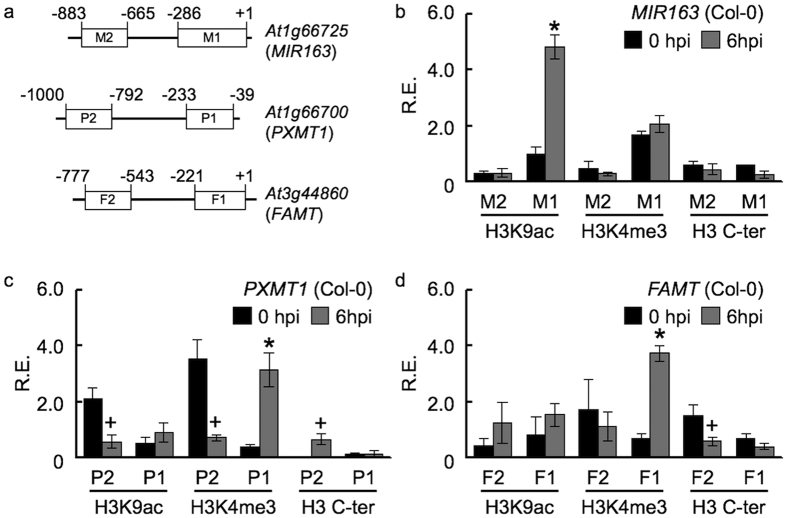
*Pst-*induced chromatin changes at the *MIR163, PXMT1* and *FAMT* loci. **(a**) Schematic diagrams of the *MIR163, PXMT1* and *FAMT* loci showing the upstream regions and target amplicons (open boxes) for ChIP DNA analyses. Positions flanking each amplicon are indicated. **(b–d)** Mature plants were treated with virulent *Pst* DC3000 and samples were collected at 0 and 6 hours post inoculation (hpi) for ChIP. Inoculation was performed using 2 × 10^8^ cfu/mL (OD_600_ = 0.4) of bacteria through dipping inoculation. Antibodies against H3K9ac, H3K4me3 and H3 C-ter were used in ChIP. Relative enrichment (R.E.) of ChIP DNA were quantified using qPCR and normalized against input DNA at the *MIR163* (**b)**, *PXMT1* **(c)** and *FAMT* **(d)** loci, respectively. Values are mean ± standard error from three biological replicates. Asterisks indicate significant differences (Student’s *t*-test; P < 0.05) when compared to the corresponding sample at 0 hpi. “+” signs indicate significant differences at P < 0.1 (Student’s *t*-test) when compared to the corresponding sample at 0 hpi.

At the *MIR163* locus, enrichment of H3K4me3 showed positive correlation with miR163 accumulation in related *Arabidopsis* polyploids. In contrast, the presence of H3K9ac is not sufficient for its active expression under normal condition^19^. Chromatin modification has been shown to play roles in controlling and priming defense gene expression under stresses^32^. Therefore, to further understand the regulation and histone modification changes at the *MIR163* and its target loci under biotic stresses, chromatin immunoprecipitation (ChIP) was used to follow the two permissive histone marks at these loci upon *Pst* DC3000 infection (Fig. 5). For qPCR, we monitored the levels of permissive histone modifications at their proximal upstream regions (M1, P1 and F1) near the transcription start site as well as a distal upstream region (M2, P2 and F2) for each locus, respectively (Fig. 5a). At the *MIR163* locus, enrichments of H3K9ac and H3K4me3 were detected at the proximal region (M1) when compared to the distal region (M2) under normal condition. Upon *Pst* DC3000 infection, H3K9ac level was enhanced (~4.9 folds) at the *MIR163* proximal upstream region (M1) while no further enrichment of H3K4me3 was detected (Fig. 5b). Using antibodies against the C-terminus of histone H3 (H3 C-ter), histone H3 occupancy at both the proximal and distal regions showed a lower level but insignificant enrichment at 6 hpi than that at 0 hpi. In contrast to the *MIR163* locus, significant enrichment of H3K4me3 at the proximal region of *PXMT1* (~8 folds) and *FAMT* (~5.5 folds) were detected upon *Pst* infection while H3K9ac levels remained unchanged at the P1 and F1 regions (Fig. 5c and d). Therefore, the increase in H3K4me3 at the *PXMT1* and *FAMT* loci correlates with their active expression upon pathogen attacks. Similar to the distal region of *MIR163*, both the P2 and F2 regions showed no significant changes of histone modifications tested upon stress, suggesting that H3K9ac and H3K4me3 levels at these regions play little roles in contributing the expression from the corresponding locus. However, it is interesting to note that the P2 region showed a general reduction in H3K9ac and H3K4me3 levels and increase in H3 occupancy upon stress, although such changes were only significant at P < 0.1 (Student’s *t-*test; Fig. 5c). No enrichment of histone modifications was detected at the *ACT7* locus before and after the pathogen attack (see Supplementary Fig. S7).

### Direct functional analyses of miR163 targets

To further characterize the direct functional roles of *PXMT1* and *FAMT* in plant defense, we overexpressed the targets under the control of the constitutive *CaMV 35S* promoter. Since the presence of endogenous miR163 could potentially affect their overexpression, silent mutations were also introduced at the miR163 complementary site in *PXMT1* and *FAMT* to decouple them from miR163 regulation, generating the c-myc-tagged *mPXMT1* and *mFAMT* for *35S-*driven overexpression in *A. thaliana*, respectively (see Supplementary Fig. S8a and b). All transgenic lines exhibited no obvious morphological changes when compared to the transgenic line carrying an empty vector control (see Supplementary Fig. S8c). To further characterize the *PXMT1* expression in the transgenic lines, two sets of primers were used to detect either the combined transcripts expression (endogenous and transgene transcripts) or the myc-tagged transgene transcripts expression by qRT-PCR. In the absence of pathogen, the combined *PXMT1* mRNA level was increased by 130-fold in the 35-PXMT1 line and by 493-folds in 35S-mPXMT1 line when compared to the vector control (see Supplementary Fig. S9a). Comparing the 35S-PXMT1 and 35S-mPXMT1 lines, expression of the myc-tagged transcripts is 4.2-fold higher in the 35S-mPXMT1 line than that in the 35S-PXMT1 line, suggesting down-regulation of the *Myc-PXMT1* by the endogenous miR163 (see Supplementary Fig. S9b). However, it should be noted that the number of transgene copies insertion and the position of the insertion locus could affect transgene expression level, leading to such difference. Regardless, these data showed that both transgenic lines showed overexpression of *PXMT1* in Col-0. When plants were inoculated with *Pst* DC3000, expression of *PXMT1* was also significantly induced at 24 hpi in the empty vector control line although the overall *PXMT1* levels were still higher in the overexpressors than in the vector control line upon the *Pst* treatment. All lines tested showed similar sensitivities to *Pst* DC3000 infection (see Supplementary Fig. S9c), suggesting a less direct correlation between the altered *PXMT1* expression and the increased resistance against *Pst* DC3000 in the *mir163* mutant. PXMT1 was recently found to associate with seed germination and early development of *Arabidopsis* seedlings^26^. It is possible that miR163 acts to repress *PXMT1* expression at the later stage of plant development.

**Figure 6:**
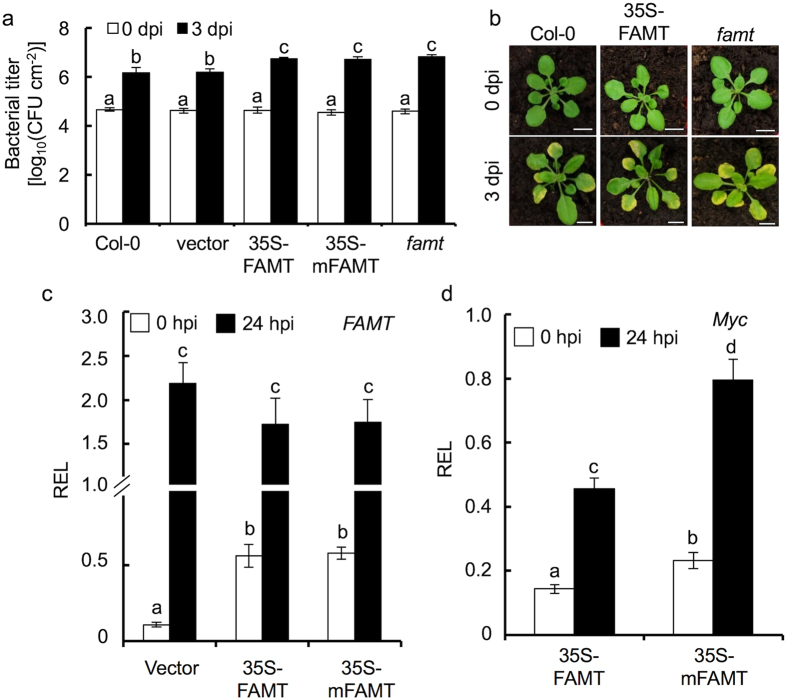
*FAMT* overexpressors and mutant are sensitive to *Pst* infection. **(a,b)** Mature plants were inoculated with *Pst* DC3000 (5 × 10^5^ cfu/mL; OD_600_ = 0.001) through syringe infiltration. (**a**) Bacterial growth in leaves was determined at 0 and 3 days post-inoculation (dpi). Error bars indicate the standard deviation from 3 replicates. Same letters denote no statistical differences among means from the replicates as calculated by ANOVA with Tukey-Kramer *post hoc* test (α = 0.05). (**b**) Plant phenotypes and disease symptoms of 4-week-old leaves at 0 and 3 dpi. Scale bar = 2 cm. Col-0, wild type *A. thaliana*; vector, transgenic line contains empty vector transgene; 35S-FAMT, transgenic line contains 35S-driven myc-tagged FAMT transgene; 35S-mFAMT, transgenic line contains the 35S-driven myc-tagged mFAMT transgene (the miR163 target site is mutated); and *famt*, the homozygous *famt* (SALK_119380) T-DNA insertion mutant. (**c,d**) Total RNA was isolated from the mature leaves at 0 and 24 hours post-inoculation (hpi). Expression of the endogenous and transgene *FAMT* transcripts **(c)** and the myc-tagged transcripts (**d**) were determined using qRT-PCR with primers targeting the gene region of *FAMT* and the *myc-*tag, respectively. The relative expression level (REL) was normalized against *EF1α* expression. Values are mean ± standard error (n = 3). Same letters denote no statistical differences (Student’s *t*-test; P < 0.05) from three biological replicates.

In the *famt* mutant, an increased sensitivity to the pathogen infection was detected when compared to the Col-0 (Fig. 6a and b). This result is in agreement with the defense response observed in the *mir163* mutant to which the *mir163* mutant showed enhanced resistance against *Pst*. In the reciprocal experiment, overexpression of FAMT is expected to confer improved resistance in the overexpressors. To our surprise, increased sensitivity to *Pst* DC3000 was also observed in the 35S-FAMT and 35S-mFAMT lines (Fig. 6a and b). Therefore, this suggested that the ectopic over-accumulation of FAMT could adversely affect the level of plant defense. In *Arabidopsis*, constitutive expression of inducible SAR resistance pathways involving the *PR1* and *NPR1* genes was found to incur costs in terms of plant size and seed yields^33^. Since the FAMT overexpressors exhibited similar increased disease sensitivity as the *famt* mutant (Fig. 6a and b), it is possible that high level of *FAMT* expression over activates the secondary metabolite production, increasing the cost burden on plant development, rendering the plant more sensitive to *Pst* infection^34^. In Col-0, *FAMT* expression showed a high basal expression under normal growth condition when compared to *PXMT1* expression (Fig. 2e)^19^. The overall *FAMT* transcript level (combined endogenous gene and transgene expression) in 35S-FAMT and 35S-mFAMT lines are higher (~5-fold) when compared to the vector control line (Fig. 6c). Such relatively small fold change of transcript expression driven by the strong *35S* promoter in the transgenic lines could be resulted from its high endogenous expression level. At 24 hpi with *Pst* DC3000, a 20-fold and 3-fold increase in *FAMT* accumulation was detected in the vector control line and the overexpressors (FAMT or mFAMT), respectively, suggesting the endogenous *FAMT* were induced at a higher level comparing to the recombinant myc-tagged transcripts to which their expressions are driven by the *35S* promoter. At the transcript level, it is expected that the 35S-mFAMT show a higher overexpression of *FAMT* due to the mutated miR163 target site when compared to the 35S-FAMT overexpressors. However, no difference in the *FAMT* expression was detected among the lines at both 0 and 24 hpi. Using the primer pair targeting the Myc-tagged transcripts (Myc-FAMT and Myc-mFAMT) in qRT-PCR, a 1.4-fold expression difference was found between the 35S-FAMT and 35S-mFAMT lines at 0 and 24 hpi (Fig. 6d), suggesting mutation at the miR163 target site had an effect in the *Myc-mFAMT* transcript accumulation. However, as mentioned above, we could not exclude the possibility that difference in transgene copies and insertion could lead to the observed difference between the 35S-FAMT and 35S-mFAMT lines. Nevertheless, since altered pathogen resistance was observed in the *famt* mutant and the *FAMT* overexpressors, these data suggested that *FAMT* contributes in stress responses against *Pst* infection.

**Figure 7:**
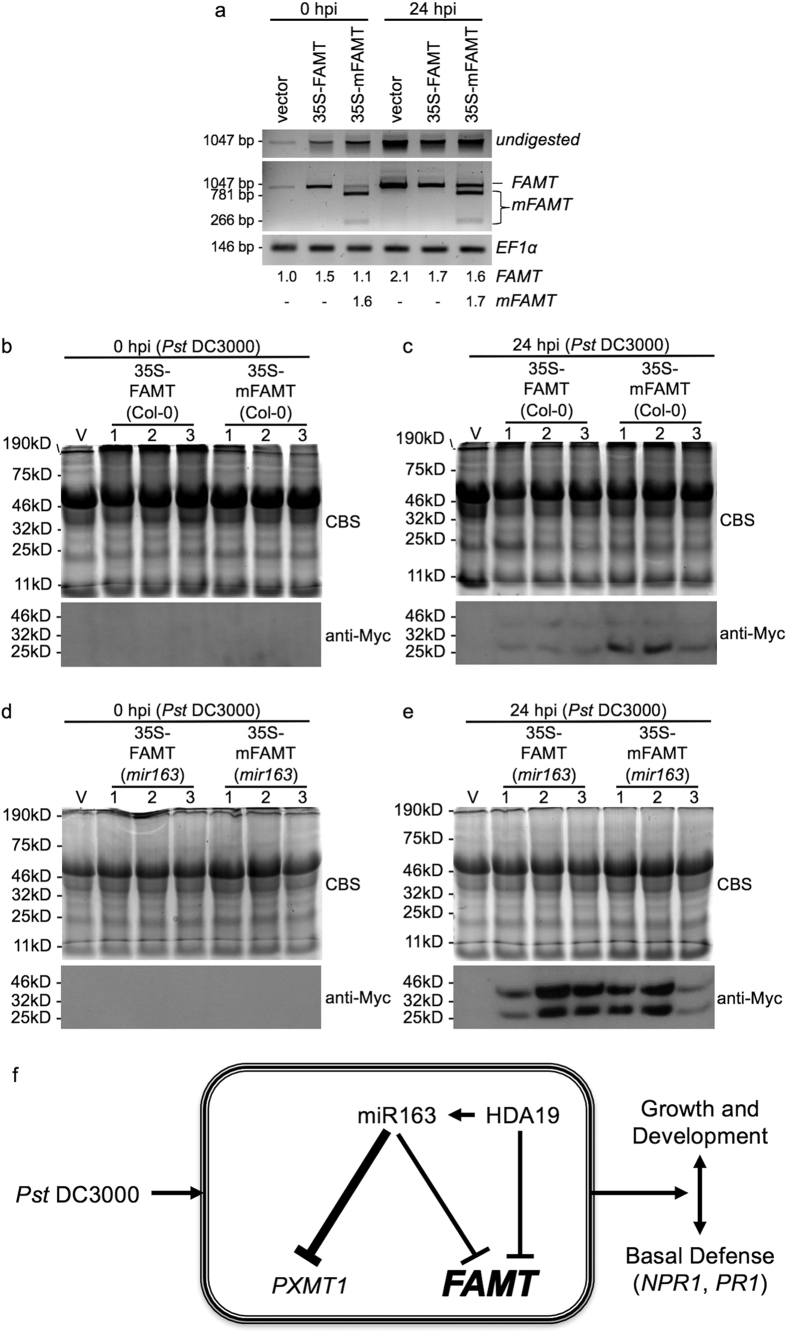
Transcript and protein accumulation in the FAMT overexpressors. **(a)** Semi-quantitative RT-PCR and cleaved amplified polymorphic sequences (CAPS) analyses were used to detect the overexpressed *FAMT* and *mFAMT* in various transgenic lines. Mature leaves were inoculated with *Pst* DC3000 (5 × 10^5^ cfu/mL) through syringe infiltration and samples were collected at 0 and 24 hpi for gene expression analyses. cDNA were digested with ApoI to determine the relative level of FAMT and mFAMT in the transgenic lines. *EF1α* expression was used as a control. Densitometry quantification of the transcripts was performed using ImageJ. The relative *FAMT* (1047 bp) and *mFAMT* (781 bp) intensities in various lines were compared against that in the vector control at 0 hpi. **(b,c)** Total leaf protein from leaves of overexpressors were extracted at 0 hpi **(b)** and after 24 hpi **(c)** upon *Pst* DC3000 treatment. Proteins were resolved in 15% SDS-PAGE for western blot analyses using antibody against the c-Myc epitope tag. CBS, Coomassie blue stained. **(d,e)** The FAMT or mFAMT overexpressor in Col-0 background was backcrossed to the *mir163* mutant. Homozygous FAMT or mFAMT overexpressor lines in the *mir163* ecotype background were selected for western blot analyses. Total leaf protein from leaves of overexpressors were extracted at 0 hpi **(d)** and after 24 hpi **(e)** upon *Pst* DC3000 treatment. Total proteins were resolved in 15% SDS-PAGE and western blot was performed using antibody against the c-Myc epitope tag. **(f)** A simplified model for miR163 and its targets regulation in plant defense. Relative thickness of lines represents the strength of activation or repression.

Since the expressed recombinant transcripts contain a diagnostic ApoI enzyme site within the mutagenized miR163 target site, this allows us to further distinguish their relative expression from the corresponding endogenous transcripts using cleaved amplified polymorphic (CAP) PCR analyses (Fig. 7a; see Supplementary Fig. S10a). Upon *Pst* infection, both the endogenous *PXMT1* and *FAMT* expression were induced. However, the elevated level of endogenous transcripts in the transgenic plants overexpressing mutated miR163 target transcripts (*mPXMT1* or *mFAMT*) was lower than that in the vector control lines, suggesting a transcriptional feedback regulation of the endogenous transcripts by the overexpressed transcripts in the overexpressors. To further verify the ectopic expression of PXMT1 and FAMT proteins in the transgenic lines, antibody against the c-myc epitope tag was used to detect the tagged-proteins in the mature leaves of the overexpressors. It is expected that mutation of the miR163 cleavage site in the 35S-mPXMT1 and 35S-mFAMT leads to high protein accumulation in the transgenic lines. However, no protein signal was detected despite higher transcript accumulation in these lines comparing to the vector control (Fig. 7a and b; see Supplementary Fig. S10), suggesting limited translation of the expressed transcripts or instability of the translated proteins.

Although *PXMT1* was ectopically expressed in the transgenic lines (see Supplementary Figs S9a,b and S10a), the inability to detect the presence of the recombinant proteins (see Supplementary Fig. S10b) could potentially attribute to the lack of physiological phenotype in defense against *Pst* (see Supplementary Fig. S9c). Interestingly, low levels of Myc-FAMT and Myc-mFAMT were detected in the overexpressors when challenged by *Pst* after 24 hpi (Fig. 7c). A doublet of proteins signal was detected, with one between 32 kD and 46 kD and the other between 25 kD and 32 kD in size. Based on the amino acid sequences, the predicted size of the myc-tagged FAMT or mFAMT is around 41 kD. Therefore, the smaller protein signal could be resulted from incomplete translation of the recombinant proteins or truncation of the translated proteins. In addition, it is possible that upon *Pst* DC3000 treatment, translation and stability of FAMT was enhanced. To test if miR163 acts to repress the translation of the target transcripts, we crossed the homozygous FAMT or mFAMT overexpressors into the *mir163* mutant background. In the *mir163* mutant background, the *FAMT* or *mFAMT* transcript accumulation was higher when compared to the vector control (see Supplementary Fig. S11). However, the increased accumulations were similar between the two overexpressors. At protein level, no recombinant proteins can be detected in the untreated plants at the *mir163* background (Fig. 7d), suggesting low level of proteins accumulation or protein instability. In the *mir163* mutant background, enhanced recombinant proteins accumulation in 35S-FAMT and 35S-mFAMT were detected when compared to that in the Col-0 background (Fig. 7e). However, no obvious difference of protein accumulation was detected among the 35S-FAMT and 35S-mFAMT overexpressors. Therefore, these data suggested that miR163 could limit the accumulation of the recombinant protein under stress conditions. Taken together, these data supported that the involvement of FAMT in plant defense is more complicated than a simple direct correlation between the miRNA and the target. Further studies are required to elucidate its exact role in plant defense against pathogen.

### A simplified model for miR163 and its targets regulation in plant defense

Based on our study, we proposed a model summarizing the role of miR163 and its regulatory relationships with its targets and their contributions to plant growth and defense (Fig. 7f). miR163 is a negative regulator involved in basal defense against plant pathogen. While miR163 acts to repress its target transcripts accumulation, different targeting efficiency was observed between the two targets (*PXMT1* and *FAMT*). In addition, histone deacetylase (HDA19) plays a role in contributing to miR163 and *FAMT* expressions but not *PXMT1* expression, suggesting multiple factors act in concert to affect both the miRNA and its targets accumulation. In plants, resource allocation during defense responses could cost burden to development and growth^35^,^36^. The production of secondary metabolites can cause extensive metabolic reprogramming, leading to costly metabolic and energy burdens on plants growth^37^,^38^. Environmental modulation of stress response through miRNAs appears to be a general mechanism in plants to prevent continual burden costs so as to retain resources for growth and development^4^,^5^. In fact, recent genome-wide analyses by Zhang *et al*.^39^ revealed that convergent evolution between miRNAs and nucleotide binding site leucine-rich repeat (*NBS-LRR*) defense genes contributes to the balance of these genes in defense and the associated fitness costs^39^. Our findings further suggested that miR163 play a modulatory role in controlling the threshold of target genes expression upon stress, thereby potentially contributing to the cost balance for establishing disease resistance while maintaining the growth of plants. In conclusion, our study has provided an example that non-conversed miRNA is induced as a part of defense response and represses its target gene expression to achieve appropriate levels of gene expression, revealing one of the evolutionary significance upon the formation and selection of new miRNA genes.

## Methods

### Plant material and growth conditions

*Arabidopsis thaliana* ecotype Columbia (Col-0) was used throughout this study. The *mir163* mutant (CS879797) was obtained from the Arabidopsis Biological Resource Center (ABRC). For plant growth, seeds were sterilized and stratified on Murashige and Skoog (Sigma-Aldrich, St. Louis, MO) media containing 3% sucrose and 0.8% agar for 2 days at 4 °C and grown at 22 °C under a 16/8 hours light/dark cycle. Two-week-old plants were transferred into new MS media about one-week prior transferred to soil. For generating *mir163 hda19* double mutant, emasculation was performed on the *mir163* mutant and followed by manual pollination with pollens from the *hda19* mutant. The obtained progenies (heterozygotes for both miR163 and HDA19) were self-fertilized to generate the segregating F2 progenies. Homozygous *mir163 hda19* double mutant (DM) were screened using PCR genotyping (see Supplementary Table S2). For plant transformation, about 5-week-old *A. thaliana* plants were used for *Agrobacterium tumefaciens*–mediated transformation through floral dipping^40^. Primary transformants (seedlings) were screened on Murashige and Skoog (MS) agar medium (Sigma-Aldrich) supplemented with corresponding antibiotics or herbicides for selection (7.5 mg/mL Basta; 50 mg/mL Kanamycin). For each transgene construct, unless otherwise specified, at least eight T1 transgenic lines were selected and screened. Stable T2 transgenic plants with 3:1 segregation resistance were then selected for further screening and analyses.

### Pathogen inoculation and enumeration

*Pseudomonas syringae* pv. *tomato* DC3000 (*Pst* DC3000) was used for the biotic stress treatment. For each genotype and sample collection, four 3–4 weeks old plants were infected with the bacteria by either syringe infiltration or dipping inoculation at ZT6^41^. Bacteria were grown overnight in LB medium at 28 °C containing 50 μg/mL kanamycin and 25 μg/mL rifampicin. Cells were harvested and washed twice. For syringe infiltration, 4–6 mature rosette leaves of similar sizes from the 4 mature plants were infiltrated with a bacterial suspension at OD_600_ = 0.001 (5.0 × 10^5^ cfu/mL cells). In dipping inoculation, the entire mature plants was dipped and submerged in a bacterial suspension at OD_600_ = 0.4 (2.0 × 10^8^ cfu/mL) in 10 mM MgCl_2_ containing 0.05% Silwet L-77 for 10 seconds. For mock infection, 10 mM MgCl_2_ was used in syringe infiltration and 10 mM MgCl_2_ with 0.05% Silwet L-77 was used in dipping treatments. Three biological replicates were performed. For bacterial enumeration, infected leaves were harvested and surface sterilized using 70% ethanol for 1 minute. Five leaf disks were excised with cork borer and macerated to release intercellular bacteria into 100 μL sterile water. Serial dilutions (from 10^−1^ to 10^−6^) of the extract were prepared. 100 μL sample from each dilution was then spread on LB plate contain 50 mg/L kanamycin and 25 mg/L rifamycin. The plates were incubated at 28 °C for 40 h and the colony-forming units (CFU) for each dilution of each sample were counted. For data processing, only plates with countable number of colonies (<250) were used to calculate the final CFU.

### Salicylic acid treatment

For salicylic acid (SA) treatment, 0.138 g SA (MW: 138.12) (Sigma-Aldrich, St. Louis, Missouri, United States) was dissolved in 1 L water, and the pH was adjusted to 5.8 using KOH. 3–4 weeks old plants grown in soil were sprayed with 1 mM SA with 0.02% Silwet L-77 (Lehle Seeds, Round Rock, Texas, United States) until the solution drips down. For each sample collection, leaves from four plants were harvested.

### Plasmid constructs

For cloning of *PXMT1 (At1g66700*) and *FAMT (At3g44860*), the cDNA were amplified using *A. thaliana* cDNA as template in PCR reactions. Oligonucleotides primers were designed to contain flanking NdeI and AvrII sites to facilitate cloning (see Supplementary Table S2). The amplified fragment was cloned into pGEM-T vector (Promega) for sequence verification. To create a 5′ Myc-tagged fusion with the cloned cDNA, a 5′ Myc-adapter (with flanking XhoI and NdeI sites) were synthesized using complimentary oligonucleotides and cloned upstream of either *PXMT1* or *FAMT* cDNA. The resulting plasmids pGEM-T/Myc-PXMT1 and pGEM-T/Myc-FAMT were used for subsequent cloning. The whole gene cassette was then released using XhoI and AvrII digestion and cloned into pEarleyGate100 vector (ABRC stock: CD3-724) to replace the gateway cassette in the vector, resulting in the 35S-driven overexpression constructs, pEG100/35S-PXMT1 and pEG100/35S-FAMT, respectively. The constructs were then individually transformed into *Agrobacterium tumefaciens* (GV3101) for plant transformation^40^. For cloning of *mPXMT1* and *mFAMT* with mutated miR163 target sites, a PCR-driven overlap extension approach^42^ was used to create the mutated *mPXMT1* and *mFAMT* cDNA with overlapping primers designed to mutagenize the target sites (see Supplementary Table S2). For PCR, the pGEM-T/Myc-PXMT1 and pGEM-T/Myc-FAMT were used as templates. The resulting spliced Myc-mPXMT1 and Myc-mFAMT from the PCR-driven overlap extension were cloned into pGEM-T vector, resulting in pGEM-T/Myc-mPXMT1 and pGEM-T/Myc-mFAMT construct respectively. Subsequently the gene cassette was released using XhoI and XbaI and cloned into pEarleyGate100 vector for plant transformation.

### RNA isolation and cDNA synthesis and quantitative real time PCR (qRT-PCR)

Mature rosette leaves before bolting (3–4 weeks old *A. thaliana*) were harvested for total RNA isolation using the Purelink Plant RNA Reagent according to the manufacturer’s protocol (Thermal Fisher Scientific, Waltham, Massachusetts, United States). After the extraction, RNA (5 μg) was treated with DNaseI (Promega, Fitchburg, Wisconsin, United States). DNaseI-treated RNA (1 μg) was reverse transcribed to cDNA using the Omniscript reverse transcription kit (Qiagen, Venlo, The Netherlands) in the presence of 25 ng/mL oligo dT (12–18) primer (GeneLink, Hawthorne, New York, United States) in a 20 μL reaction according to the manufacturer’s instructions. The products were diluted to a final volume 150 μL with H_2_O for qPCR and 1 μL cDNA was used for qRT-PCR. A negative control without RNA was included. For qRT-PCR, SYBR green PCR Master Mix (Roche, Upper Bavaria, Germany) was used in the qRT-PCR system and fluorescent signals were detected using 7500 Real Time PCR System (Applied Biosystems, Foster City, California, United States). Primers used for qPCR were listed in Supplementary Table S3. Expression levels of genes were normalized using *EF1*-α as the endogenous control. Statistical analysis was determined with three independent biological replications by either two-tailed Student’s *t*-test (p < 0.05) or ANOVA with Tukey-Kramer *post hoc* test (α = 0.05) as stated in the text.

### Small RNA gel blot analysis

Total RNA (10 μg) was resolved on a denaturing 15% polyacrylamide gel and transferred to a Hybond N+ membrane (GE Healthcare, Piscataway, New Jersey, United States). After ultraviolet (UV) crosslinking, the membrane was prehybridized in Church buffer^43^ at 40 °C for 1 h. Oligonucleotide complementary to miR163 was end labeled with [γ-^32^P] ATP (6000 Ci/mmol) using T4 polynucleotide kinase (NEB, Ipswich, Massachusetts, United States) and purified using a Costar Spin-X centrifuge tube column containing G-25 Sephadex (Sigma-Aldrich). Oligonucleotide complementary to the antisense U6 oligonucleotide was also end-labeled using [γ-^32^P] ATP (150 Ci/mmol). For hybridization, the small RNA blot was incubated overnight at 40 °C in the presence of radioactive labeled probes for miR163 or U6^19^. After hybridization, the blot was rinsed twice with 2x standard saline citrate (2xSSC) containing 0.5% sodium dodecyl sulfate (SDS) for 10 mins at 42 °C, then washed with 0.5×SSC containing 0.1% SDS solution for 15 min at 42 °C. Small RNA hybridization signals were detected by exposing the blot to an X-ray film. For image development, Carestream^®^ Kodak^®^ autoradiography GBX developer and fixer (Kodak, Rochester, New York, United States) were used. Densitometric intensity was quantified using ImageJ software (National Institutes of Health, Bethesda, Maryland, United States). To prevent saturation of target signals, separate exposure times were used for miR163 and U6 detection.

### Cleaved amplified polymorphic sequences (CAPS) analysis

4 μL cDNA was used in a 50 μL PCR reaction containing 1 μL each of the gene-specific primers (see Supplementary Table S4), 1 μL 10 mM dNTPs, 5 μL 10x ThermoPol buffer and 0.25 μL Taq DNA polymerase. After PCR, 20 μL PCR product was digested using *ApoI*. As an undigested control, 20 μL PCR product was used in a parallel reaction without the enzyme. Reactions were incubated at 50 °C overnight (16 hours) and the products were resolved in 2% agarose gel and visualized by SYBR-Safe staining. The relative expression levels of endogenous transcript and miR163-resistant transcripts (*mFAMT, mPXMT1*) in digested PCR product were quantified using ImageJ software.

### Protein extraction and western blot

For western blot analyses, five independent transgenic lines were tested for the recombinant protein accumulation and data from representative lines were selected for presentation. In each biological replicate, mature leaves from nine plants were collected for protein extraction. Total leaf protein was extracted in leaf protein extraction buffer (50 mM Tris-HCL pH8, 150 mM NaCl, 1 mM EDTA, 10% glycerol, 1% Trition X-100 and 2 mM PMSF), followed by centrifugation at 12,000 g for 5 mins at 4 °C. The supernatant was collected and total protein was quantitated by Bradford assay (BioRad, Hercules, California, United States) and 80 μg protein samples were subjected to 15% SDS-PAGE. Two parallel gels were prepared for total leaf protein separation. As an internal control for loading of the SDS-PAGE gel, a gel was stained with coomassie blue after electrophoresis. Parallel gel was electroblotted onto PVDF membrane (Amersham, Little Chalfont, UK) for immunoblotting analysis. Membranes were blocked overnight at 4 °C in 5% non-fat milk in PBS-T (10 mM Na_2_HPO_4_, 137 mM NaCl, 2.7 mM KCl, 1.8 mM KH_2_PO_4_ and 0.05% Tween 20). After blocking, membranes were incubated for 2 h at room temperature with primary antibodies against the c-Myc epitope tag (Invitrogen, Carlsbad, California, United States) at 1:5000 dilution in PBS-T with 5% non-fat milk, then followed by washing two times for 10 mins with PBS-T. For secondary antibodies incubation, blots were further incubated for 2 h at room temperature with Goat anti-Mouse IgG (H + L) secondary antibody, HRP-conjugate (Invitrogen) at 1:2000 dilution in PBS-T with 5% non-fat milk. Protein signals were detected on medical X-ray film using the supersignal west pico chemiluminescent substrate kits (ThermoFisher Scientific) following manufacturer’s instructions. For PXMT1 and mPXMT1 overexpressors, western blots were performed five times with similar results. For FAMT and mFAMT overexpressors, western blots were performed twice with similar results.

### Chromatin immunoprecipitation (ChIP) and qPCR

3 to 4 weeks old rosette leaves (2 g) from Col-0 and the *mir163* mutant before and 6 hours after treatment with virulent *Pst* DC3000 were harvested and placed under vacuum in 37.5 mL crosslinking buffer (0.4 M sucrose, 10 mM Tris, pH 8, 1 mM EDTA, 1 mM PMSF, and 1% formaldehyde). After 10 mins, 0.125 M glycine (2.5 mL 2 M Glycine in 37.5 mL) was used to stop the crosslinking, followed by freezing and grinding of the samples using liquid nitrogen. The grounded tissues were homogenized in nuclei isolation buffer (0.25 M sucrose, 15 mM PIPES, pH 6.8, 5 mM MgCl_2_, 60 mM KCl, 15 mM NaCl, 1 mM CaCl_2_, 0.9% Triton X-100, 1 mM PMSF, 1X ProBlock™ Gold Plant Protease Inhibitor Cocktail [Gold Biotechnology]) and filtered through 3 layers of miracloth, the filtrate was then centrifuged at 11,000 g for 20 mins at 4 °C. The nuclear pellet was resuspended in nuclei lysis buffer (50 mM HEPES, pH 7.5, 150 mM NaCl, 1 mM EDTA, 1% Triton X-100, 0.1% deoxycholate, 0.1% SDS, 1 mM PMSF, 1X ProBlock™ Gold Plant Protease Inhibitor Cocktail). The chromatin was subjected to sonication (10 pulses, high power for 10 times) using Bioruptor plus (Diagenode) to obtain 200–600 bp DNA fragments. The sonicated chromatin was then centrifuged at 13,800 g for 10 mins at 4 °C to remove cell debris. Immunoprecipitation (IP) was performed using 750 μL sonicated chromatin with antibodies against the C-terminus of histone H3 (H3 C-ter, 2 μg; ab1791; Abcam, Cambridge, Massachusetts, United States), H3K4me3 (4 μg; ab8580; Abcam, Cambridge, Massachusetts, United States), or H3K9ac (3.6 μg; ab10812; Abcam, Cambridge, Massachusetts, United States). Mock controls without antibodies are included in the IP reaction. IP was performed using Dynabeads^®^ Protein A (Invitrogen, Carlsbad, California, United States) as described in the manufacturer’s instructions. DNA from IP was resuspended in 300 μL TE (pH 8). The pull down immune-complex and input DNA (sonicated chromatin without IP) were then subjected to reverse cross-linked at 65 °C under high salt (0.2 M NaCl) conditions for 8 h. After samples were subjected to proteinase K digestion to remove proteins and DNA was then purified by phenol-chloroform extraction and ethanol precipitation. Finally, the purified DNA was resuspended in 21 μL TE (pH8). For gene enrichment analysis, purified DNA from was diluted 10 times and input DNA control was diluted 20 times, 1 μL diluted-DNA was subjected to qPCR using primers shown in Supplementary Table S4.

## Additional Information

**Accession codes:** Sequence data from this article can be found in the GenBank/EMBL data libraries under the following accession numbers: *MIR163 (At1g66725*), *PXMT1 (At1g66700*), *FAMT (At3g44860*), *NPR1 (At1g64280*), *PR1 (At2g14610*), *PDF1.2 (At5g44420*), *PAD3 (At3g26830*), *EF1-α (At1g07930*), *Actin7 (At5g09810*).

**How to cite this article**: Chow, H.T. and Ng, D.W-K. Regulation of miR163 and its targets in defense against *Pseudomonas syringae* in *Arabidopsis thaliana. Sci. Rep.* **7**, 46433; doi: 10.1038/srep46433 (2017).

**Publisher's note:** Springer Nature remains neutral with regard to jurisdictional claims in published maps and institutional affiliations.

## Supplementary Material

Supplementary Information
