# Supplementary material for: Regulation of miR163 and its targets in defense against Pseudomonas syringae in Arabidopsis thaliana
Source: Sci Rep. 2017 Apr 12;7:46433. doi: 10.1038/srep46433 (PMC5388894; doi:10.1038/srep46433)
Supplement: Supplementary Information [file srep46433-s1.pdf]

Supplementary Information

Regulation of miR163 and its targets in defense against *Pseudomonas syringae* in *Arabidopsis thaliana*

Hiu Tung Chow[1,2,†] and Danny Wang-Kit Ng[1,2,*]

[1]Department of Biology, Hong Kong Baptist University, Kowloon Tong, Hong Kong, China

[2]The Partner State Key Laboratory of Agrobiotechnology, The Chinese University of Hong Kong, Shatin, Hong Kong, China

[†]Present address: School of Life Sciences and State Key Laboratory of Agrobiotechnology, The Chinese University of Hong Kong, Shatin, Hong Kong, China

*Address correspondence to dannyng@hkbu.edu.hk (D.W-K.N.)

The following materials are available in the online version of this article.

Supplementary Methods

Supplementary Figures S1 to S11

Supplementary Tables S1 to S4

SUPPLEMENTARY METHODS

In silico promoter analysis

Cis-element motifs at the upstream regions of *MIR163* (-1439/-1), *PXMT1* (-2500/-1) and *FAMT* (-2700/-1) were identified using the PlantCARE [1] and PLACE [2] online. These elements were grouped into different functional categories and the numbers of elements presence were counted for each upstream region (Table S1).

Luciferase (LUC) reporter constructs

For luciferase (LUC) reporter constructs, the 1.5 kb *ProPXMT1* (-1406/+68) and 1.2 kb *ProFAMT* (-1124/+56) promoter containing the upstream sequence (relative to the transcription start site) and the 5' untranslated region (5'UTR) were amplified using *A. thaliana* genomic DNA as template and cloned into pGEM-T vector (Promega) for sequence verification. The cloned promoter was then fused upstream of the luciferase (LUC) reporter, through AatII and XhoI sites in the pFAMIR plasmid that was modified from pFGC5941 [3]. The resulting fusion constructs were then transformed into *Arabidopsis* to create the ProPXMT1-LUC and ProFAMT-LUC reporter lines, respectively. A promoterless-LUC construct was included as a vector only control.

Luciferase assay

The luciferase activity of transgenic lines containing ProFAMT-LUC or ProPXMT1-LUC transgene was analyzed using a luminometer (TECAN Infinite M200). A total 14 ProPXMT1-LUC and 16 ProFAMT-LUC transgenic lines were used. For each transgenic line, eight 2-week-

old seedlings were transferred to OptiPlate-96 white plates (PerkinElmer) containing MS agar with 30 g/L sucrose. 30 μL of 0.5 mM luciferin (Gold Biotechnology, Olivette, Missouri, United States) was then added into each well and incubated for 1h before measurement. For signal detection, luminescence signals were integrated for a period of 100. Signals from at least 14 independent lines were measured and presented as means ± SE. The average of a promoterless-LUC transgenic line was used for normalization.

REFERENCES

1. Rombauts S, Déhais P, Van Montagu M, Bouzé P. PlantCARE, a plant *cis*-actingregulatory element database. *Nucleic Acids Res* **27**, 295-296 (1999).

2. Higo K, Ugawa Y, Iwamoto M, Korenaga T. Plant *cis*-acting regulatory DNA elements (PLACE) database: 1999. *Nucleic Acids Res* **27**, 297-300 (1999).

3. McGinnis K*, et al.* Transgene-induced RNA interference as a tool for plant functional genomics. *Methods in Enzymology* **392**, 1-24 (2005).

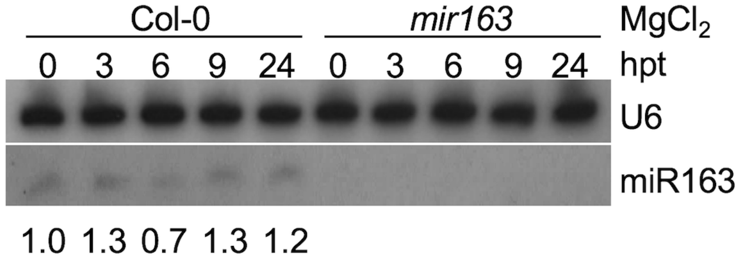

Figure S1. miR163 accumulation under control treatment. Temporal miR163 accumulation at 0, 3, 6, 9 and 24 hpi in Col-0 and the *mir163* mutant upon 10 mM $MgCl_2$ with 0.02% Silwet L-77 treatment (control). The corresponding U6 signals (endogenous controls) were detected in the same blot. Densitometric analysis was performed using ImageJ software and the miR163 signals were normalized against U6. The relative fold change of miR163 was showed at the bottom. Experiments were performed twice with similar results.

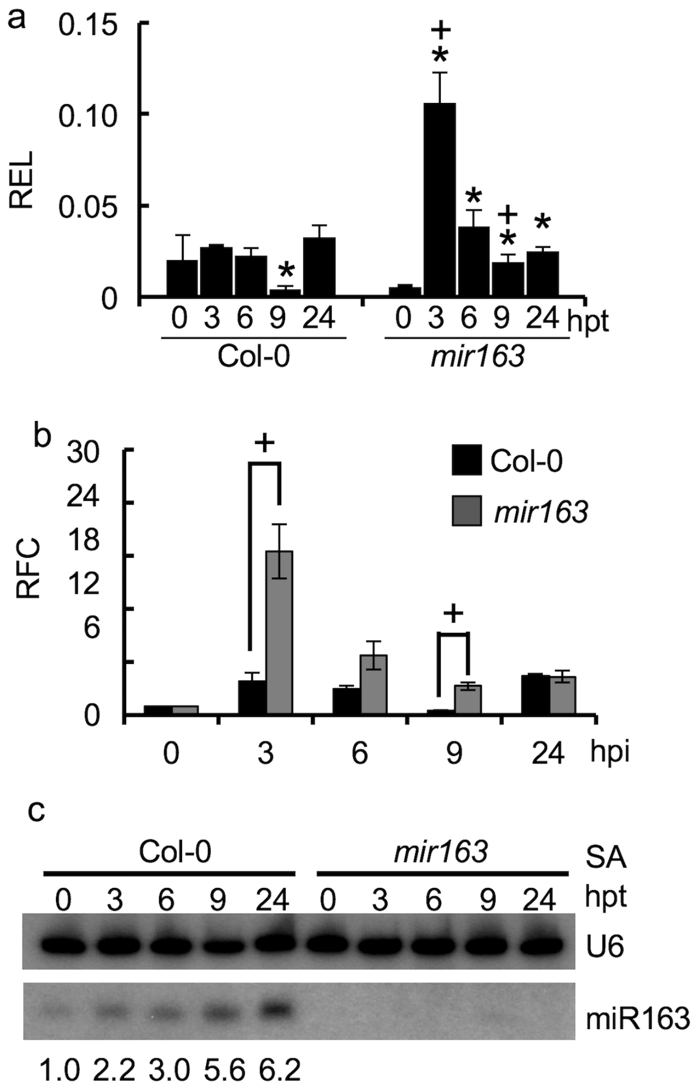

Figure S2. Effects of SA on *pri-miR163* and miR163 expression.
(a-b) Expression of *pri-miR163* in Col-0 and the *mir163* mutant before (0) and at 3, 6, 9, 24 hours post-treatment (hpt) with 1mM salicylic acid (SA) by spraying. **(a)** The relative expression level (REL) of *pri-miR163* was calculated using *EF1α* as a control. **(b)** Upon induction, the relative fold change (RFC) of pri-miR163 at different time point was compared to that at 0 hpi. Values are mean ± standard error (n = 3). Asterisks indicate significant differences (Student's *t*-test; $P < 0.05$) between 0 hpi and the indicated time point within the genotype. "+" signs indicate significant differences (Student's *t*-test; $P < 0.05$) between Col-0 and the *mir163* at the indicated time point.
(c) Temporal miR163 accumulation at 0, 3, 6, 9 and 24 hpt in Col-0 and the *mir163* mutant upon SA treatment was detected using small RNA gel blot analysis. The corresponding U6 signals (endogenous controls) were detected in the same blot. Densitometric analysis was performed using ImageJ software and the miR163 signal was normalized against U6. The SA-induced fold change of miR163 was showed at the bottom. Experiments were performed twice with similar results.

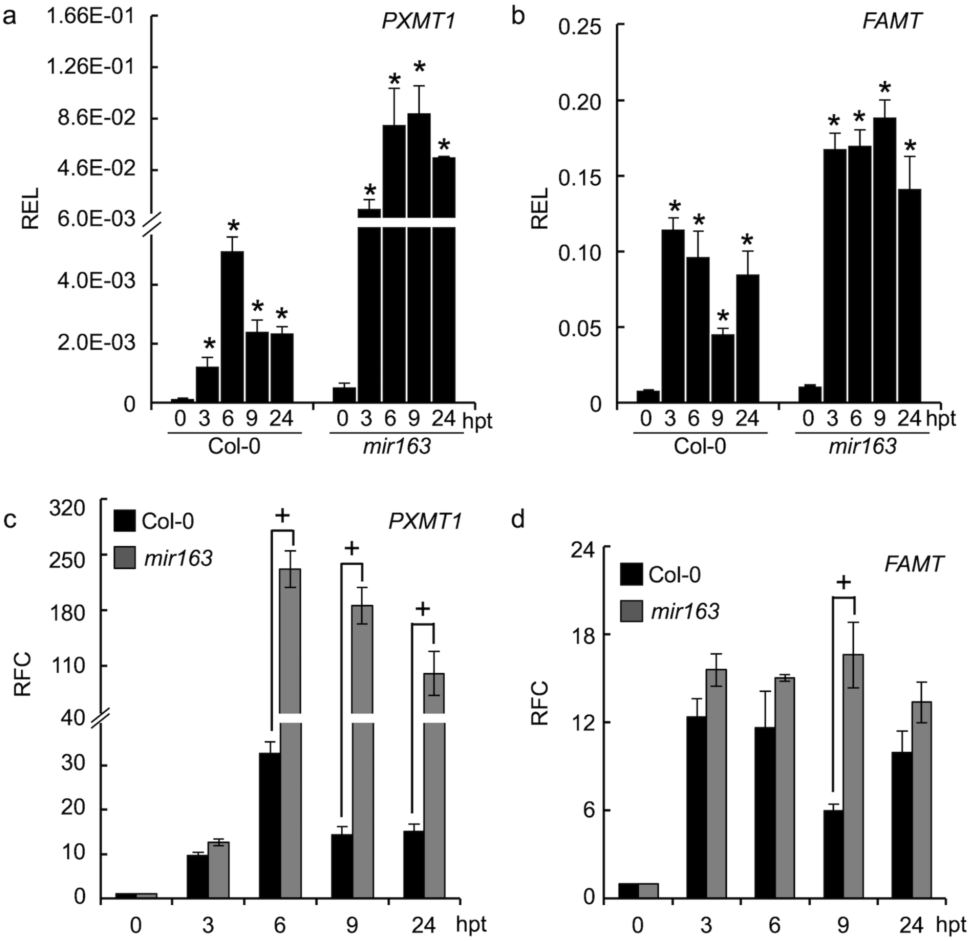

Figure S3. Effects of SA on miR163 targets expression.
(a-b) Expression of miR163 targets in Col-0 and the *mir163* mutant before (0) and at 3, 6, 9, 24 hours post-treatment (hpt) with 1mM salicylic acid (SA) by spraying. **(a)** The relative expression level (REL) of *PXMT1* **(a)** and FAMT **(b)** were calculated using *EF1α* as a control. **(c-d)** Upon induction, the relative fold change (RFC) of *PXMT1* **(c)** and FAMT **(d)** at different time point was compared to that at 0 hpi. Values are mean ± standard error (n = 3). Asterisks indicate significant differences (Student's *t*-test; P < 0.05) between 0 hpi and the indicated time point within the genotype. "+" signs indicate significant differences (Student's *t*-test; P < 0.05) between Col-0 and the *mir163* at the indicated time point.

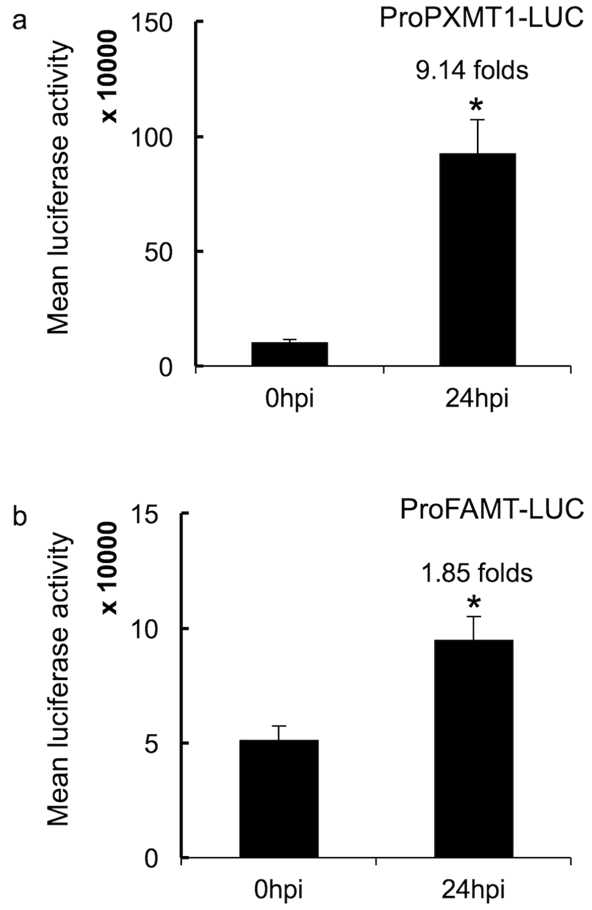

Figure S4. ***Pst*-induced ProPXMT1-LUC and ProFAMT-LUC expression**

(a) Bioluminescence counts (in ten thousands [10000]; y axis) for ProPXMT1-LUC expression in two-week-old seedlings before inoculation (0) and at 24 hours post-inoculation (hpi) with *Pst* DC3000. Inoculation was performed using $2x10^8$ cfu/mL of bacteria through flooding inoculation. Error bars indicate the standard error from multiple independent transgenic lines (n = 14). Asterisk denotes statistical significant difference (Student *t*-test, P < 0.05) compared to seedlings before inoculation.

(b) Bioluminescence counts (in ten thousands [10000]; y axis) for ProFAMT-LUC expression in two-week-old seedlings before inoculation (0) and at 24 hpi with virulent *Pst* DC3000. Inoculation was performed as in **(a)**. Error bars indicate the standard error from multiple independent transgenic lines (n = 16). Asterisk denotes statistical significant difference (Student *t*-test, P < 0.05) compared to seedlings before inoculation.

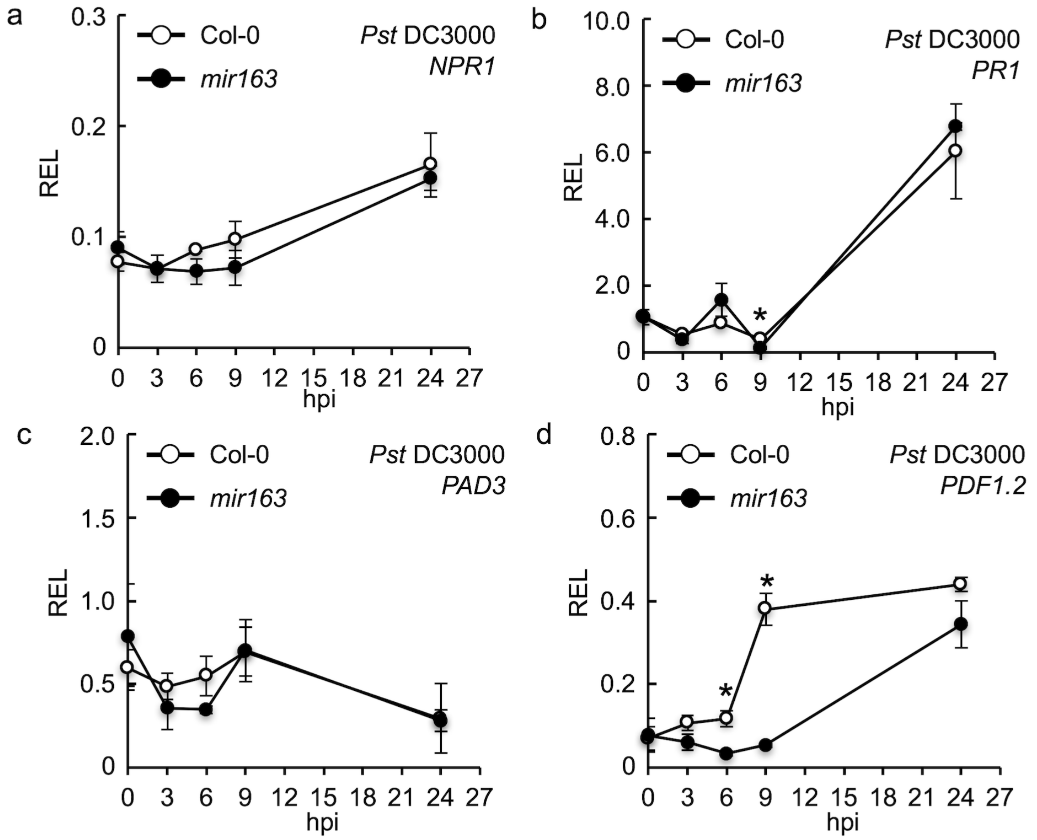

Figure S5. Induction of plant defense responsive gene expression in the Col-0 and *mir163* mutant during *Pst* DC3000 infection.

Expression of *NPR1* **(a)**, *PR1* **(b)**, *PAD3* **(c)** and *PDF1.2* **(d)** in Col-0 and the *mir163* mutant before inoculation (0) and at 3, 6, 9, 24 hours post-inoculation (hpi) with virulent *Pst* DC3000. Inoculation was performed using 2 x 10^8 cfu/mL (OD_{600} = 0.4) of bacteria through dipping inoculation. The relative expression level (REL) was normalized against *EF1α* expression. Values are mean ± standard error (n = 3). Asterisks indicate significant difference between 0 hpi and the indicated time using Student's *t*-test ($P < 0.05$).

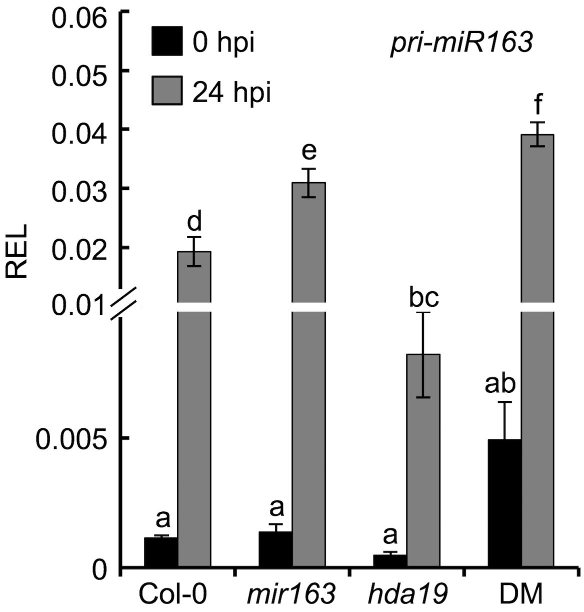

Figure S6. Expression of *pri-miR163* in the *hda19* mutant under biotic stresses.
Wild type (Col-0), the *mir163* mutant (CS879797), the *hda19* mutant (SALK_139445) and homozygous *mir163hda19* double mutant (DM) were infiltrated with a suspension of *Pst* DC3000 (OD_{600} = 0.001 in 10mM $MgCl_2$; 5 x 10^5 cfu/mL). Expression of *pri-miR163* was detected using qRT-PCR at 0 and 24 hours post-inoculation (hpi). The relative expression level (REL) was normalized against *EF1α* expression. Values are mean ± standard error (n = 3). Same letters denote no statistical differences among means as calculated by ANOVA with Tukey-Kramer *post hoc* test (α = 0.05).

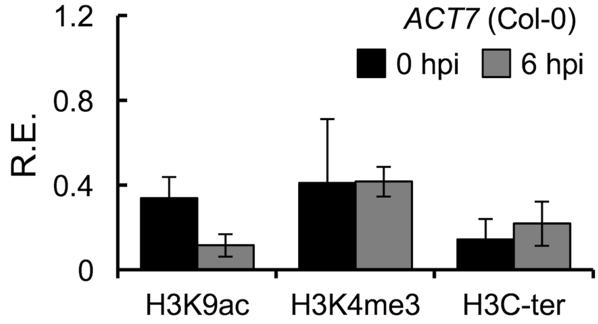

Figure S7. Histone modifications at the *Actin7* locus.
Mature plants were treated with virulent *Pst* DC3000 and samples were collected at 0 and 6 hours post inoculation (hpi) for ChIP. Inoculation was performed using 2 x 10^8 cfu/mL (OD_{600} = 0.4) of bacteria through dipping inoculation. Antibodies against H3K9ac, H3K4me3 and H3C-ter were used for ChIP. Relative enrichment (R.E.) of ChIP DNA were quantified using qPCR and normalized against input DNA. Primers targeting the +713/+968 region of *ACT7* (*At5g09810*) were used. Values are mean ± S.E. from three biological replicates.

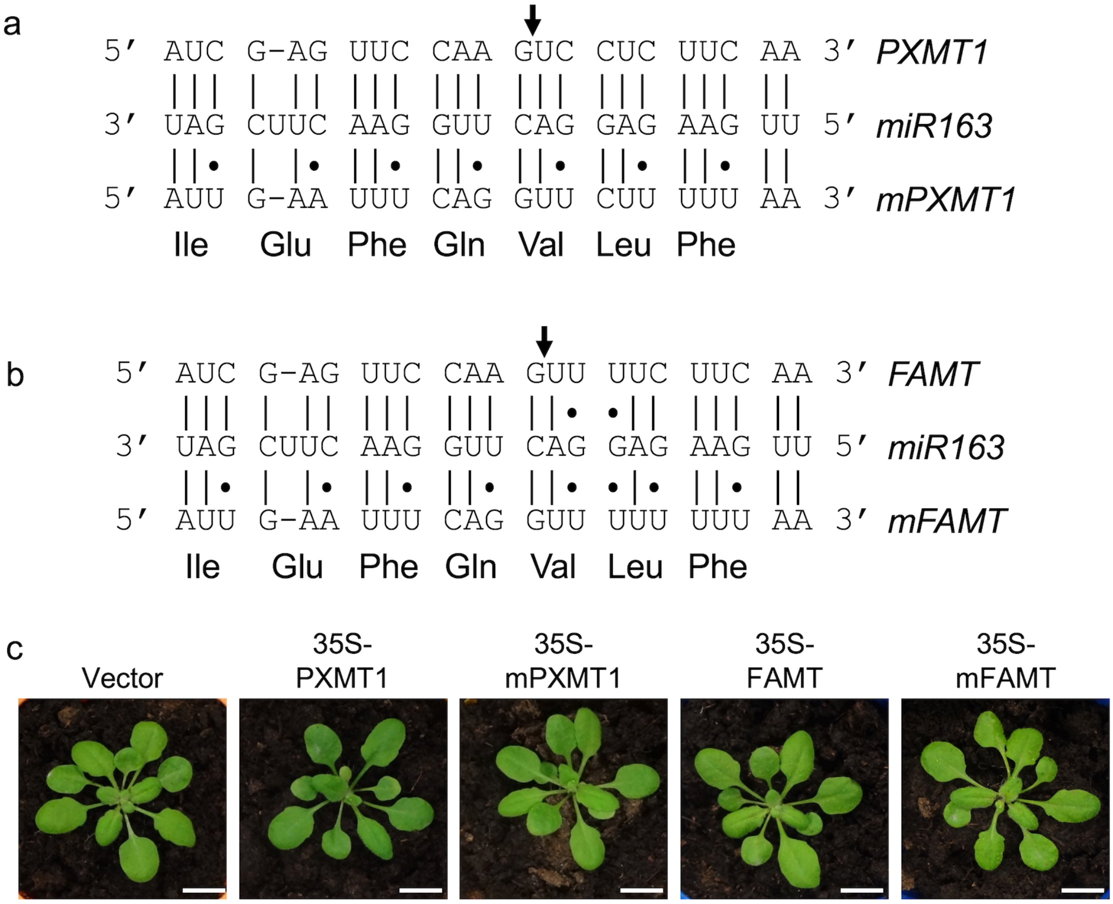

Figure S8. Mutated miR163 recognition sequences in the miR163 target overexpressors and their phenotypes.

(a-b) Core sequence alignment of miR163 against its targets, *PXMT1* **(a)** and *FAMT* **(b)**. The miR163 cleavage sites in *PXMT1* and *FAMT* are indicated by an arrow, respectively. Silent mutations were introduced at the miR163 target recognition site in *PXMT1* and *FAMT* cDNA, generating the *mPXMT1* and *mFAMT* cDNA respectively. Corresponding encoded amino acid residues are showed at the bottom. Individual cDNA of the wild type and the mutated miR163 targets was then fused with a 5' c-Myc epitope tag and expressed under the control of the *CAMV 35S* promoter in transgenic *Arabidopsis thaliana*.

(c) Representative photos of 26-day-old rosette of transgenic lines carrying different transgene constructs were showed. Vector, empty vector alone; 35S-PXMT1, 35S-driven myc-tagged *PXMT1* transgene; 35S-mPXMT1, 35S-driven myc-tagged *mPXMT1* transgene; 35S-FAMT, 35S-driven myc-tagged *FAMT* transgene; and 35S-mFAMT, 35S-driven myc-tagged *mFAMT* transgene. Scale bar = 1.5 cm.

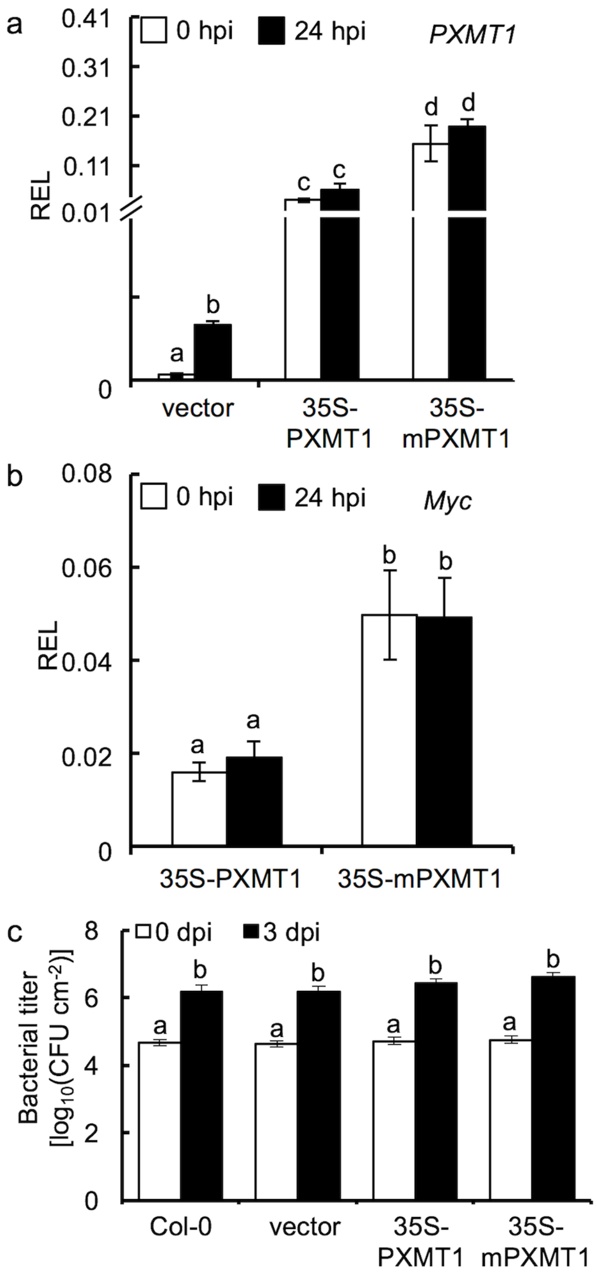

Figure S9. *PXMT1* overexpression has no effect on pathogen sensitivity.

(a-b) Mature plants were inoculated with *Pst* DC3000 (5 x 10^5 cfu/mL; OD_{600} = 0.001) through syringe infiltration. Total RNA was isolated from the mature leaves at 0 and 24 hours post-inoculation (hpi). Expression of the endogenous and transgene *PXMT1* transcripts **(a)** and the myc-tagged transcripts **(b)** were determined using qRT-PCR with primers targeting the gene region of *PXMT1* and the *myc*-tag, respectively. The relative expression level (REL) was normalized against *EF1α* expression. Values are mean ± standard error (n = 3). Same letters denote no statistical difference (Student's *t*-test; $P < 0.05$).

(c) Bacterial growth in leaves were determined at 0 and 3 days post-inoculation (dpi). Error bars indicate the standard deviation from 3 replicates. Same letters denote no statistical differences among means as calculated by ANOVA with Tukey-Kramer *post hoc* test (α = 0.05) from three biological replicates. Col-0, wild type *A. thaliana*; vector, transgenic line contains empty vector transgene; 35S-PXMT1, transgenic line contains 35S-driven myc-tagged PXMT1 transgene; and 35S-mPXMT1, transgenic line contains the 35S-driven myc-tagged mPXMT1 transgene (the miR163 target site is mutated).

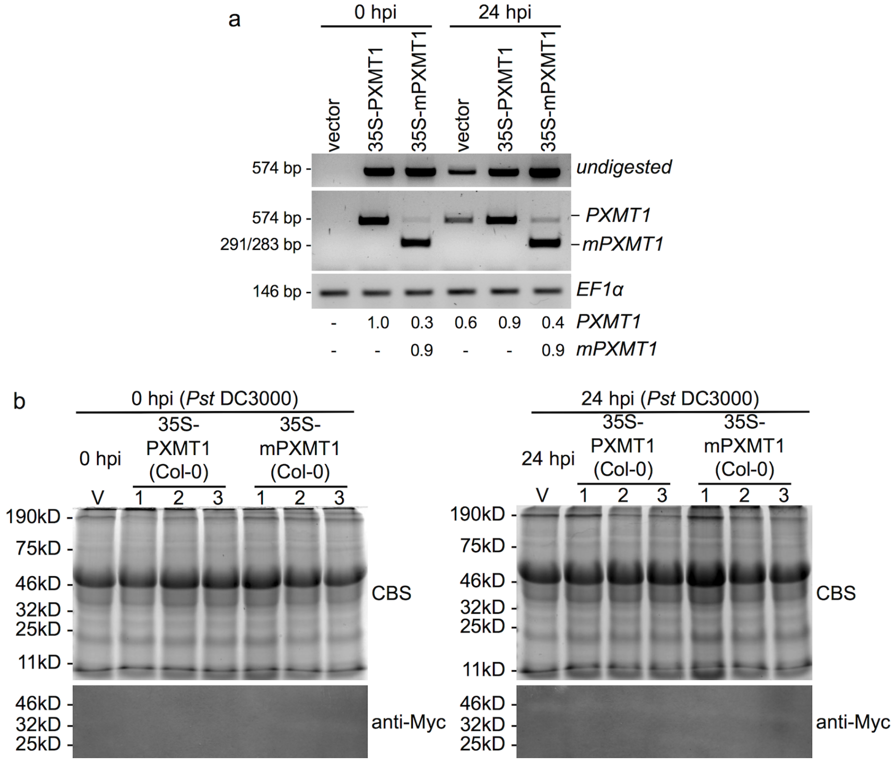

Figure S10. Transcript and protein accumulation in the PXMT1 overexpressors.
(a) Semi-quantitative RT-PCR and cleaved amplified polymorphic sequences (CAPS) analyses were used to detect the overexpressed *PXMT1* and *mPXMT1* in various transgenic lines. Mature leaves were inoculated with *Pst* DC3000 (5 x 10^5 cfu/mL) through syringe infiltration and samples were collected at 0 and 24 hpi for gene expression analyses. cDNA were digested with ApoI to determine the relative level of *PXMT1* and *mPXMT1* in the transgenic lines. *EF1α* expression was used as a control. Densitometry quantification of the transcripts was performed using ImageJ. The relative *PXMT1* (547bp) and *mPXMT1* (291/283bp) intensities in various lines were compared against that in the 35S-PXMT1 line at 0 hpi.
(b) Total leaf protein was extracted from various lines following treatment as in **(a)** and resolved in 15% SDS-PAGE. Western blot was performed using antibody against the c-Myc epitope tag. CBS, Coomassie blue stained.

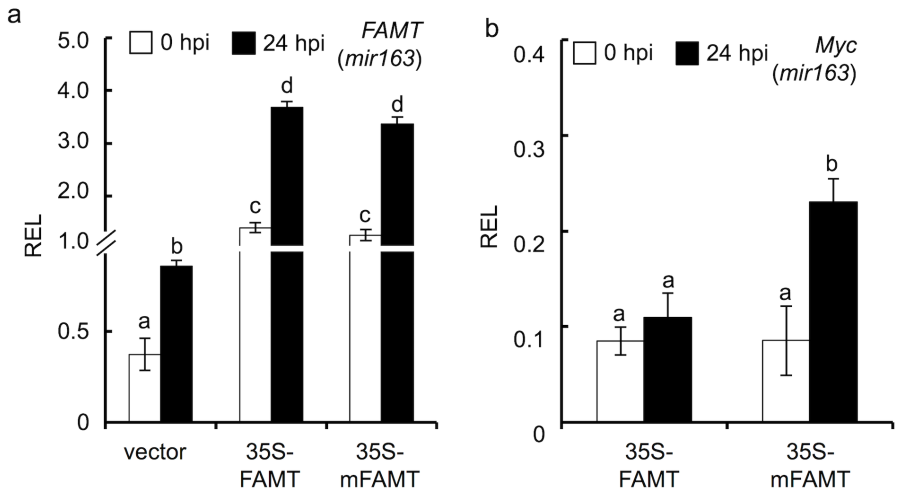

Figure S11. Overexpression of *FAMT* in the *mir163* mutant

Mature plants were inoculated with *Pst* DC3000 (5 x 10^5 cfu/mL; $OD_{600} = 0.001$) through syringe infiltration. Total RNA was isolated from the mature leaves at 0 and 24 hours post-inoculation (hpi). Expression of the endogenous and transgene *FAMT* transcripts **(a)** and the myc-tagged transcripts **(b)** were determined using qRT-PCR with primers targeting the gene region of *PXMT1* and the *myc*-tag, respectively. The relative expression level (REL) was normalized against *EF1α* expression. Values are mean ± standard error (n = 3). Same letters denote no statistical difference (Student's *t*-test; $P < 0.05$) from three biological replicates.

Supplementary Table S1. Putative pathogen responsive cis-elements at the upstream regions of MIR163 and its target

Category and *cis*-element[1]	Accession (Gene)	*At1g66700* (*PXMT1*)								*At1g66725* (*MIR163*)						*At3g44860* (*FAMT*)									
	Position relative to TSS	-2500/-1501		-1500/-1001		-1000/-501		-500/-1		-1439/-1001		-1000/-501		-500/-1		-2700/-2001		-2000/-1501		-1500/-1001		-1000/-501		-500/-1	
	Strand / Consensus[3]	+	-	+	-	+	-	+	-	+	-	+	-	+	-	+	-	+	-	+	-	+	-	+	-
**Promoter consensus**																									
TATA box	TATAAA	7	10	3	4	4	2	10	9	1	2		5	3	4	1	2	8	2	-	1	3	7	10	10
CAAT box	GGCCAATCT	-	-	-	-	-	-	-	-	1	2	3	4	2	3	2	3	-	1	-	-	2	1	-	2
ACGTC BOX (bZIP factor binding)	GACGTC	-	-	-	-	-	-	-	-	-	-	-	-	-	-	-	-	-	-	-	-	-	-	-	-
Other positive regulatory element[2]	CAACA, NGATT, TAACTG	9	7	3	4	4	5	3	4	-	3	-	6	6	4	5	7	3	4	2	6	6	5	5	2
**Biotic stress responsive elements**																									
ASF1MOTIFCAMV (salicylic acid response)	TGACG	1	-	-	-	-	-	-	-	-	-	1	-	1	-	-	-	-	-	-	-	-	1	-	1
CACGTGMOTIF (PR gene responsive element)	CACGTG	1	-	-	1	-	-	-	-	-	-	-	-	-	-	-	-	-	-	-	-	-	-	-	-
GT1CONSENSUS (GT-1 binding site)	GRWAAW	4	2	5	1	5	6	2	5	2	-	3	3	2	4	-	-	1	2	3	2	6	-	2	2
MYB1LEPR (PR gene responsive element)	GTTAGTT	-	-	-	-	-	-	-	-	-	-	-	-	1	-	-	-	-	-	-	-	1	-	-	-
WBBOXPCWRKY1 (W-box)	TTTGACY	1	1	-	-	1	-	-	-	-	-	-	-	-	-	-	-	-	-	-	-	-	-	-	-
WBOXATNPR1 (salicylic acid-induced WRKY)	TTGAC	2	1	-	-	2	-	-	-	1	-	1	-	-	-	2	2	-	1	3	2	-	-	-	3
I-box	GATAAG	1	-	2	-	-	-	-	-	1	-	2	-	-	-	1	2	1	1	-	2	-	-	2	-
Evening element	AAAATATCT	-	-	-	-	-	1	-	-	-	-	-	-	-	-	-	-	-	-	-	-	-	-	1	-
**Elicitor responsive elements**																									
ABRE (Abscisic acid response)	ACGTG	-	-	2	-	-	-	-	-	-	-	-	-	1	-	-	-	-	-	-	-	-	-	-	2
DPBFCOREDCDC3 (Abscisic acid response)	ACACNNG	1	1	2	2	-	-	-	-	-	-	-	-	1	-	-	-	-	1	1	1	-	-	-	-
LTRECOREATCOR15 (Abscisic acid response)	CCGAC	-	-	-	-	-	-	-	-	-	-	-	-	-	-	-	-	-	-	1	-	-	-	-	-
MYBATRD22 (Abscisic acid response)	CTAACCA	-	-	-	-	-	-	-	-	-	-	-	-	-	-	-	1	-	-	-	-	-	1	-	-
MYCATRD22 (Abscisic acid response)	CACATG	-	-	-	-	-	-	-	-	-	-	-	-	-	-	-	-	1	2	1		-	-	-	-
MYCCONSENSUSAT (Abscisic acid response)	CANNTG	-	-	-	-	-	-	-	-	2	2	1	1	3	3	-	-	3	3	2	2	-	-	-	-
ARF/ ARFAT (Auxin response)	TGTCTC	-	-	-	-	-	-	-	-	-	-	-	-	-	1	-	-	1	1	-	1	-	-	-	-
**Abiotic stress responsive elements**																									
MBS (drought-inducibility)	CAGTTG	-	1	-	-	-	-	-	-	-	-	-	-	-	-	-	-	-	-	1	-	-	-	-	-
ANAERO2CONSENSUS (Anaerobic stress)	ACGACG	-	-	-	-	-	-	-	-	-	2	2	-	-	-	-	-	1	-	-	-	-	-	-	-
MYBCORE (Water stress)	CNGTTR	3	-	-	2	-	-	-	1	1	-	2	-	-	-	-	-	-	-	-	-	-	-	-	-
ACGTATERD1 (Dought)	ACGT	3	3	1	1	1	1	1	1	-	1	-	-	1	1	-	1	-	-	-	1	-	2	-	-
MYB2CONSENSUSAT (Dehydration-responsive)	YAACKG	-	1	1	-	-	-	-	-	-	-	-	-	-	-	-	-	3	3	2	2	-	-	-	-
**Light responsive elements**																									
TCT-motif	TCTTAC	-	-	-	1	-	-	1	-	-	-	-	-	-	-	-	-	-	-	-	-	-	1	-	-
IBOXCORE	GATAA	1	1	4	1	1	-	2	2	2		2	2	-	-	1	-	1	3	-	2	-	-	2	-
GATA-motif	WGATAR	2	2	4	2	3	1	2	1	4	4	2	4	1	1	5	4	1	2	1	2	2	-	4	2
GAG-motif	AGAGAGT	-	-	-	-	-	-	-	-	-	-	-	-	-	-	-	-	-	-	-	-	-	-	-	-
**Specificity elements**																									
RHERPATEXPA7 (Root)	KCACGW	-	-	1	2	1	-	-	-	-	-	-	1	-	-	-	-	-	-	-	-	-	-	2	-
UP2ATMSD (Axillary bud)	AAACCCTA	-	-	-	-	-	-	-	-	-	-	-	1	-	-	-	-	-	-	-	-	-	-	-	-
SURECOREATSULTR11 (Root)	GAGAC	-	-	3	-	-	-	-	-	-	-	-	-	1	1	1	1	-	-	-	-	-	-	-	-

[1] Upstream regions of miR63 and its targets were analyzed using the PlantCARE (Rombauts et al., 1999) and PLACE (Higo et al., 1999) online.
The numbers of putative *cis*- elements identified within a specific region were indicated.

[2] Include RAV1AAT (CAACA), ARR1AT (NGATT) and MYB2AT (TAACTG)

[3] N = A, T, C or G; R = A or G; Y = C or T; W = A or T; K = G or T

Supplementary Tables

Supplementary Table S2. Oligonucleotides used for PCR genotyping and cloning

Name	Sequence (5' - 3')*	Target(s)
At-miR163+989-R	cctaggCAAATCAAGCGTCCAGAC	*mir163* genotyping
pDAP101-LB3	TAGCATCTGAATTTCATAACCAATCTCGATACAC	*mir163* genotyping
LBb1.3-2	cggtcATTTTGCCGATTTCGGAAC	SALK lines genotyping
SALK_139445-LP	ACTCTCTTCCTTGTCTGCGTG	*hda19* genotyping
SALK_139445-RP	ACCAGACAATGAATCAGCACC	*hda19* genotyping
LP-SALK_119380	CGAGTCACGGTCTTTGATTTC	*famt* genotyping
RP -SALK119380	ATGCTCAACACCATGAAAACC	*famt* genotyping
XhoI-Myc-adapter-F[1]	tcgagcccATGGCGGAGGAACAGAAACTGATCTCCG AAGAAGATCTGca	Myc-tagged linker
NdeI-Myc-adapter-R[1]	tatgCAGATCTTCTTCGGAGATCAGTTTCTGTTCCT CCGCCATgggc	Myc-tagged linker
NdeI-PXMT1-F[2]	catATGACTACTACTCCAGATTGGATCATGA	*At1g66700* cDNA
AvrII-PXMT1-R[2]	cctaggTTAGTTCTTGCGAAGCACG	*At1g66700* cDNA
NdeI-FAMT-F[2]	catATGTCGACTTCATTCACAATGATCGGC	*At3g44860* cDNA
AvrII-FAMT-R[2]	cctaggTCAGTTCCTTCGAAGCACAATG	*At3g44860* cDNA
XhoI-Myc-F[3]	ctcgagCCCATGGCGGA	Myc
mPXMT-R[3]	TTaAAaAGaACcTGaAAtTCaATGTTATCTGCCGGG TTTTGTC	*At1g66700* (miR163 target site mutated)
mPXMT-F[4]	ATtGAaTTtCAgGTtCTtTTtAATGATTTCAGCCTCA ATGAT	*At1g66700* (miR163 target site mutated)
XbaI-PXMT1-R[4]	tctagaCTCACCTAGGTTAGTTCTTGCGAA	*At1g66700*
mFAMT-R[3]	TTaAAaAAAACcTGaAAtTCaATTCCCTCAATATTA CTTTCT	*At3g44860* (miR163 target site mutated)
mFAMT-F[4]	ATtGAaTTtCAgGTTTTtTTtAATGATTCTTCAAACA ACGAT	*At3g44860* (miR163 target site mutated)
XbaI-FAMT-R[4]	TCTAGACTCACCTAGGTCAGTTCCTTCG	*At3g44860*
AatII-1406PXMT1-F[5]	gacgtcCAATCCTCCAAATAAAAATGAGAGC	*At1g66700* promoter
XhoI+68PXMT1-R[5]	ctcgagGATATCTCTCTCTCTTTTTTCTCAA	*At1g66700* promoter
AatII-1124FAMT-F[5]	gacgtcAGAACACATGTTTAGGCAT	*At3g44860* promoter
XhoI+56FAMT-R[5]	ctcgagGTCTCTCTTTTAACTCTGGTCT	*At3g44860* promoter

*Small case letters indicate the sequence with added flanking restriction enzyme sites in the gene-specific primer or introduced internal mutations

[1]Oligonucleotides for creating an XhoI-Myc-NdeI adapter

[2]Oligonucleotide primers for cloning of expression constructs

[3]Oligonucleotide primers for cloning of expression constructs with introduced mutated miR163 target sites; primers amplified the 5' overlapped template fragment

[4]Oligonucleotide primers for cloning of expression constructs with introduced mutated miR163 target sites; primers amplified the 3' overlapped template fragment

[5]Oligonucleotide primers for cloning the -1406/+68*PXMT1* and -1124/+56*FAMT* promoter regions

Supplementary Table S3. Oligonucleotides used for gene expression analyses

Name	Sequence (5' - 3')	Target(s)
95-AtMIR163-F	GAGCATAGGTCTTGATTGGTGGAAGACA	*At1g66725*
185-AtMIR163-R	GTCGTTGAAGAGGTTGGAACTCGATT	*At1g66725*
83PXMT-F	GATTGGAGGAGACGGTCCTGAGA	*At1g66700*
267PXMT-R	GGCTGAGATCGCCTTGGTCAT	*At1g66700*
174FAMT-F	TCCTCTGGACCGAACACTTTCAC	*At3g44860*
322FAMT-R	GTCTTGAAGAGAGTGTTAAAATCGTTGTTTGAAG	*At3g44860*
1319EF1α-F	GACATGAGGCAGACTGTTGCA	*At1g07930*
1381EF1α-R	CCGGTTGGGTCCTTCTTGT	*At1g07930*
Myc-02-F	CTCGAGCCCATGGCG	*Myc-tag*
Myc-02-R	GCAGATCTTCTTCGGAGATCAGT	*Myc-tag*
NPR1-1424-F	TGAAGATGACGCTGCTCGATCTT	*At1g64280*
NPR1-1522-R	CCCTTCATTTCGGCGATCTCCATT	*At1g64280*
50PR1-F	TAGGTGCTCTTGTTCTTCCCTCGA	*At2g14610*
155PR1-R	TCCCACTGCATGGGACCTA	*At2g14610*
1PDF1.2-F	ATGGCTAAGTTTGCTTCCATCATCAC	*At5g44420*
123PDF1.2-R	TGACCATGTCCCACTTGGCT	*At5g44420*
803PAD3-F	TGATGATCGATATGAAGAAGAAGCAAGAGAA	*At3g26830*
988PAD3-R	TCTCGTCTTGCACTTTCTTCATCACTCT	*At3g26830*

Supplementary Table S4. Oligonucleotides used for ChIP-PCR and CAPS analyses

Name	Sequence (5' - 3')	Target(s)
-286MIR163-F[1]	CGGCCAATGCGTATCCACTAGT	*At1g66725* (M1)
+1MIR163-R[1]	GATAATCCATGGACGCCTCTATGCTTAT	*At1g66725* (M1)
-39-PXMT1-F[1]	AGTGATAATCCATGGACCTCTCGATG	*At1g66700* (P1)
-233-PXMT1-R[1]	CGCAATGCCGTCACTTATAATTTGTCA	At1g66700 (P1)
-221-FAMT-F[1]	GTCTTTTATTGGCACGTTTTGTGGATGC	*At3g44860* (F1)
+1-FAMT-R[1]	TGAATTTTTAGTTGGAAGACTTCGTGCTT	*At3g44860* (F1)
-883-MIR163-F[2]	TTGTGATGGTGTTGGTAAAGACGA	*At1g66725* (M1)
-680-MIR163-R[2]	GTCATTTTATGAAATGGTGAGCTAAGTCAC	*At1g66725* (M1)
-792-PXMT1-F[2]	TGGATCAAGCATAGCGGATCAAAT	*At1g66700* (P1)
-1000-PXMT1-R[2]	CCAAAAAACTTAATAGTGTGCTATTTATGGGA	At1g66700 (P1)
-735-FAMT-F[2]	GAGATAGTGGCCTAATGGTTGAGTG	*At3g44860* (F1)
-543-FAMT-R[2]	TCATTTCAAACATGTCGTTAACTTTCCGTTA	*At3g44860* (F1)
ACT7-F[3]	ATGGCCGATGGTGAGGATATTCAG	*At5g09810*
ACT7-R[3]	CATCTTTCTGACCCATACCAACCATGA	*At5g09810*
1-PXMT1-CAPS-F[4]	CGATGACTACTACTCCAGATTGGATCATGAT	*At1g66700*
574-PXMT1-CAPS-R[4]	TGGTGTCGATAGAGTACTGGTCAAGA	*At1g66700*
1-FAMT-CAPS-F[4]	CGCATGTCGACTTCATTCACAATGATCG	*At3g44860*
1047-FAMT-CAPS-R[4]	TCAGTTCCTTCGAAGCACAATGAGG	*At3g44860*

[1]Oligonucleotide primers for ChIP-qPCR, amplifying the proximal regions of target promoters
[2]Oligonucleotide primers for ChIP-qPCR, amplifying the distal egions of target promoters
[3]Oligonucleotide primers for ChIP-qPCR, amplifying the *Actin7* control
[4]Oligonucleotide primers for CAPS analyses, amplifying both the endogenous and ectopically expressed targets
